# The *Burkholderia cenocepacia* iron starvation σ factor, OrbS, possesses an on-board iron sensor

**DOI:** 10.1093/nar/gkac137

**Published:** 2022-03-02

**Authors:** Aaron T Butt, Christopher D Banyard, Sayali S Haldipurkar, Kirsty Agnoli, Muslim I Mohsin, Srdjan Vitovski, Ameya Paleja, Yingzhi Tang, Rebecca Lomax, Fuzhou Ye, Jeffrey Green, Mark S Thomas

**Affiliations:** Department of Infection, Immunity and Cardiovascular Disease, Faculty of Medicine, Dentistry and Health, University of Sheffield, Sheffield S10 2RX, UK; Department of Infection, Immunity and Cardiovascular Disease, Faculty of Medicine, Dentistry and Health, University of Sheffield, Sheffield S10 2RX, UK; Department of Infection, Immunity and Cardiovascular Disease, Faculty of Medicine, Dentistry and Health, University of Sheffield, Sheffield S10 2RX, UK; Department of Infection, Immunity and Cardiovascular Disease, Faculty of Medicine, Dentistry and Health, University of Sheffield, Sheffield S10 2RX, UK; Department of Infection, Immunity and Cardiovascular Disease, Faculty of Medicine, Dentistry and Health, University of Sheffield, Sheffield S10 2RX, UK; Department of Infection, Immunity and Cardiovascular Disease, Faculty of Medicine, Dentistry and Health, University of Sheffield, Sheffield S10 2RX, UK; Department of Infection, Immunity and Cardiovascular Disease, Faculty of Medicine, Dentistry and Health, University of Sheffield, Sheffield S10 2RX, UK; Department of Infection, Immunity and Cardiovascular Disease, Faculty of Medicine, Dentistry and Health, University of Sheffield, Sheffield S10 2RX, UK; Department of Infection, Immunity and Cardiovascular Disease, Faculty of Medicine, Dentistry and Health, University of Sheffield, Sheffield S10 2RX, UK; Section of Structural Biology, Department of Infectious Disease, Imperial College London, Sir Alexander Fleming Building, South Kensington, London SW7 2AZ, UK; School of Biosciences, University of Sheffield, Sheffield S10 2TN, UK; Department of Infection, Immunity and Cardiovascular Disease, Faculty of Medicine, Dentistry and Health, University of Sheffield, Sheffield S10 2RX, UK

## Abstract

*Burkholderia cenocepacia* is an opportunistic pathogen that causes severe infections of the cystic fibrosis (CF) lung. To acquire iron, *B. cenocepacia* secretes the Fe(III)-binding compound, ornibactin. Genes for synthesis and utilisation of ornibactin are served by the iron starvation (IS) extracytoplasmic function (ECF) σ factor, OrbS. Transcription of *orbS* is regulated in response to the prevailing iron concentration by the ferric uptake regulator (Fur), such that *orbS* expression is repressed under iron-sufficient conditions. Here we show that, in addition to Fur-mediated regulation of *orbS*, the OrbS protein itself responds to intracellular iron availability. Substitution of cysteine residues in the C-terminal region of OrbS diminished the ability to respond to Fe(II) *in vivo*. Accordingly, whilst Fe(II) impaired transcription from and recognition of OrbS-dependent promoters *in vitro* by inhibiting the binding of OrbS to core RNA polymerase (RNAP), the cysteine-substituted OrbS variant was less responsive to Fe(II). Thus, the cysteine residues within the C-terminal region of OrbS contribute to an iron-sensing motif that serves as an on-board ‘anti-σ factor’ in the presence of Fe(II). A model to account for the presence two regulators (Fur and OrbS) that respond to the same intracellular Fe(II) signal to control ornibactin synthesis and utilisation is discussed.

## Graphical Abstract

**Graphical Abstract:**
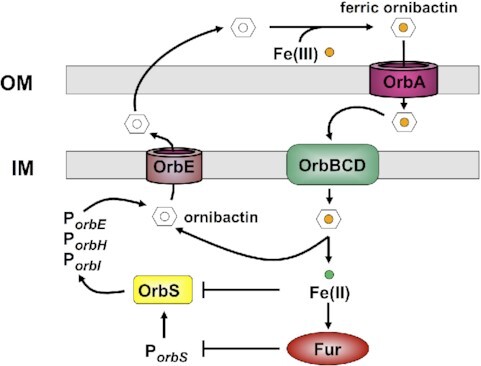
The iron-sensing domain of OrbS acts as an on-board anti-σ factor to switch off synthesis of ornibactin before intracellular iron accumulates to toxic levels.

## INTRODUCTION

Transcription is the fundamental biological process that permits the information stored in DNA to be expressed. RNA polymerase (RNAP) uses one strand of a DNA duplex as a template to synthesize complementary RNA molecules (transcripts), which include mRNA, rRNA, tRNA and regulatory RNAs that perform a variety of essential cellular functions. Bacterial RNAP is a multi-subunit enzyme with a core catalytic structure (core RNAP) consisting of five subunits (α_2_ββ'ω) that is capable of sustaining transcription (1). However, in order to locate where transcription should begin, the core RNAP requires an additional subunit, the sigma factor (σ). Binding of σ to core RNAP produces RNAP holoenzyme (holoRNAP) that can recognise and initiate transcription from specific DNA sequences (promoters) (2,3). The use of alternative σ factors, which recognise different promoter sequences, is one of several mechanisms that allow bacteria to regulate gene expression in response to environmental conditions (4).

The σ^70^ family is the largest group of bacterial σ factors, and members have a conserved modular structure consisting of up to four domains (5). Members of the σ^70^ family have been classified into four groups. Groups 1 and 2 consist of primary (housekeeping) and related σ factors; Group 3 σ factors regulate processes such as flagella biosynthesis, sporulation, and heat shock responses; and Group 4 consists of the extracytoplasmic function (ECF) σ factors (6). The ECF σ factors were first identified by virtue of their regulatory roles. They differ from the archetypal σ^70^ proteins in possessing only two (domains 2 and 4) of the four conserved domains of canonical primary σ^70^ proteins (5). Domain 2 contains motifs (regions 2.2–2.4) that are required for recognition of the -10 element and domain 4 contains region 4.2 for binding to the -35 element of cognate promoters (5,7–10). In the absence of a signal, canonical ECF σ factors are sequestered by membrane-anchored anti-σ factors, thereby preventing association with core RNAP. In these cases, promoter recognition and transcription of ECF-regulated gene(s) are dependent on receipt of an appropriate stimulus being detected by the C-terminal extracytoplasmic domain of the anti-σ factor (reviewed by (6)). On receipt of a signal, the anti-σ factor is typically degraded, allowing the release of the σ factor and recruitment by core RNAP for subsequent transcription of the output genes (11–13).

*Burkholderia* is a genus containing approximately 30 species of gram-negative bacteria which includes the *B. cepacia* complex (BCC) (14). BCC members, such as *B. cenocepacia*, are notorious for causing infections of the lungs of cystic fibrosis (CF) patients, often resulting in a necrotizing pneumonia that can lead to fatal cepacia syndrome (15). They are also associated with infections of patients with chronic granulomatous disease and in individuals who are compromised for other reasons (16). Due to their high level of intrinsic resistance to many antibiotics, eradication of BCC bacteria is challenging (17–19).

To establish an infection in the CF lung, *B. cenocepacia* must deploy mechanisms to acquire iron when challenged by host systems that restrict the availability of iron (20–22). Thus, BCC bacteria secrete high affinity, low molecular weight Fe(III)-binding compounds known as siderophores. The Fe(III)-siderophore complexes are then imported through siderophore-specific, TonB-dependent outer membrane transporters (TBDTs) (reviewed by (23–25)). Most BCC species produce the tetrapeptide siderophore, ornibactin, under iron-limiting conditions, while a subset of BCC species, including *B. cenocepacia*, also produce pyochelin (25–29). Animal infection models and ‘omics studies have demonstrated the importance of these siderophores in pathogenesis (26,30–35). However, although iron is an essential nutrient for most pathogenic bacteria, including *B. cenocepacia*, excess iron is toxic because it promotes the production of damaging reactive oxygen species (36). Therefore the process of iron acquisition by *B. cenocepacia* must be precisely regulated.

The production and transport of ornibactin in *B. cenocepacia* is coded for by a cluster of 15 genes (29). The products of the *orbG*-*orbL*, *pvdA* and *pvdF* genes are involved in the synthesis and assembly of ornibactin. These include the non-ribosomal peptide synthetases (NRPSs) OrbI and OrbJ that assemble the tetrapeptide backbone (29). The assembled siderophore is then exported, most probably through OrbE (23). Upon binding exogenous Fe(III), the Fe(III)-ornibactin complex is transported across the outer membrane into the periplasmic space through the TBDT, OrbA (33). The products of the *orbB*, *orbC* and *orbD* genes are predicted to constitute an ABC transporter that translocates Fe(III)-ornibactin across the cytoplasmic membrane into the cytoplasm whereupon bound iron is reductively released by the *orbF* gene product (23,29). These genes are organised into three transcription units, each of which is under the control of single promoters (*P_orbE_*, *P_orbH_* and *P_orbI_*) that are served by the iron starvation (IS) ECF σ factor, OrbS (29,37). In common with many IS σ factors, transcription of *orbS* is regulated in response to the prevailing iron concentration through the action of Fur (the ferric uptake regulator) at the σ^70^-dependent *orbS* promoter, *P_orbS_* (Supplementary Figure S1A; 29,38–40). Thus, under conditions of iron sufficiency, intracellular Fe(II) binds to the Fur protein and the resulting holo-repressor docks with the Fur box overlapping *P_orbS_*, thereby inhibiting transcription of *orbS* (29,41).

The well-characterized *P. aeruginosa* IS ECF σ factor, PvdS, is, overall, very similar to OrbS and is activated by proteolysis of the anti-σ factor FpvR when ferripyoverdine binds to its cognate TBDT, FpvA (42–44). This mechanism of signal transduction, involving translation of a signal perceived at the surface of the bacterium into IS σ-dependent gene expression, has been referred to as cell surface signalling (CSS) (43,45,46). However, because the ornibactin TBDT, OrbA, lacks the N-terminal domain that in FpvA triggers the PvdS-activating proteolytic cascade, and inactivation of the *orbA* gene did not impair ornibactin production, it was considered unlikely that OrbS was part of a CSS system (23,29,32). Furthermore, unlike the majority of IS ECF σ factors, OrbS is not encoded adjacent to an anti-σ factor gene, and no evidence has been obtained to suggest that OrbS is regulated by an anti-σ factor (29). For this reason, it was proposed that the observed down-regulation of OrbS-dependent gene transcription and inhibition of ornibactin biosynthesis that occurs under iron sufficient conditions was largely a consequence of decreased intracellular abundance of OrbS as a result of Fur-mediated repression of *P_orbS_* (29). Here we show that despite the absence of an anti-σ factor-mediated mechanism for regulating OrbS activity, OrbS itself is subject to a post-translational regulatory mechanism. However, unlike CSS systems, where the activity of the IS σ factor is regulated in response to the extracellular availability of the corresponding Fe(III)-siderophore complex, OrbS responds to the intracellular iron pool through possession of a C-terminal iron-sensing domain that serves as an on-board anti-σ factor.

## MATERIALS AND METHODS

### Biological resources

Bacterial strains and plasmids are listed in Supplementary Tables S1 and S2, respectively, and are available on request.

### Media and growth conditions

*B. cenocepacia* strains were routinely cultured at 37°C on M9 salts agar containing 0.5% (w/v) glucose as carbon source (47). For some experiments involving *B. cenocepacia* broth cultures, the M9 medium was also supplemented with 0.1 or 0.25% casamino acids (M9-CAA), as indicated. Lysogeny broth (LB; tryptone 10 g/l, yeast extract 5 g/l, NaCl 10 g/l) and EB medium (tryptone 3.3 g/l, yeast extract 1.7 g/l, NaCl 5 g/l) were also used, as indicated. *Escherichia coli* strains were cultured at 37°C in LB or on LB agar. Iso-Sensitest medium (IST; Oxoid) was used when trimethoprim was included in the medium and Lennox medium (tryptone 10 g/l, yeast extract 5 g/l, NaCl 5 g/l) when selecting for kanamycin and tetracycline resistance. Antibiotics were used at the following concentrations unless otherwise stated: ampicillin, 100 μg/ml (*E. coli*); trimethoprim, 25 μg/ml (*E. coli* and *B. cenocepacia*); chloramphenicol, 25 μg/ml (*E. coli*) and 50 μg/ml (*B. cenocepacia*) for LB medium and 100 μg/ml for M9 medium (*B. cenocepacia*); kanamycin, 50 μg/ml (*E. coli* and *B. cenocepacia*); tetracycline, 10 μg/ml (*E. coli*), 125 μg/ml (*B. cenocepacia*); gentamicin, 25 μg/ml (*E. coli*). For strains carrying pEX18-derived plasmid constructs, 5-bromo-4-chloro-3-indolyl-β-D-galactopyranoside (X-gal) at 40 μg/ml and isopropyl β-D-1-thiogalactopyranoside (IPTG) at 0.1-0.2 mM were included in solid agar for blue/white screening. All liquid cultures were grown at 180 rpm for aeration, unless otherwise stated. CAS agar was made by adding 10 ml of chrome azurol-sulphonate (CAS) reagent (0.605 mg/ml CAS, 0.729 mg/ml HDTMA, 100 μM FeCl_3_ (standard iron concentration) or 600 μM FeCl_3_ (high iron)) to 90 ml Y minimal agar (1.87 mg/ml glutamic acid, 3.33 mg/ml Tris base, 100 μl of 10% (w/v) MgSO_4_·7H_2_O, 100 μl of 22% (w/v) CaCl_2_·6H_2_O and 100 μl of 22% (w/v) K_2_HPO_4_·3H_2_O (pH 6.8) per 100 ml) (29). To establish iron starvation conditions, 2,2′-dipyridyl was added to M9-CAA medium and LB at final concentrations of 100 μM and 200 μM, respectively, for *B. cenocepacia* or at a final concentration of 175 μM for *E. coli* cultures (LB). For iron replete conditions, iron (III) chloride was added to a final concentration of 50 μM (except for high iron CAS agar where it was 60 μM).

### Reagents

The sequences of oligonucleotides used in plasmid construction are listed in Supplementary Table S3. Biolayer interferometry (BLItz) analysis was performed using the BLItz System (fortéBIO, Pall Corp., USA) with BLItz Pro 1.1 software in the Advanced Kinetics mode and Ni-NTA Biosensors (fortéBIO, Pall Corp., USA).

### Plasmid construction

Plasmids were introduced into *E. coli* by transformation (48) and into *B. cenocepacia* by conjugation (49,50). pKAGd4-P_orbHds96_ was constructed by annealing complementary oligodeoxynucleotides porbH-fwd-HindIII and porbH-rev-BamHI, that include nucleotides at positions -43 to +6 of the *orbH* promoter (Supplementary Figure S1B), and then ligating the double-stranded oligonucleotide to the HindIII and BamHI sites of pKAGd4. Due to the first two bases of the BamHI site corresponding to those at positions +7 and +8 of *P_orbH_*, the promoter extends to position +8. Annealing of oligonucleotides was performed by incubating the oligonucleotide pair at 90°C for 10 min in a volume of 100 μl containing 45 μl of each oligonucleotide (100 μM) and 10 μl water, and then transferring the mixture to room temperature for 60 min. 1 μl of a tenfold dilution of this mixture (4.5 μM double-stranded oligonucleotide) was used for the ligation reaction.

pBBR5-orbS was made by transferring the 0.97 kb HindIII-BamHI *orbS* fragment from pBBR1MCS-orbS to the corresponding sites of pBBR1MCS-5.

Cysteine to alanine codon substitutions were introduced into *orbS* as follows. To generate the *orbS*-C196A and -C199A alleles, *orbS* was amplified from pBBR1MCS-orbS with primer orbSfor in combination with Cysala1rev (for the C196A codon substitution) or Cysala2rev (for the C199A codon substitution) and the PCR products were digested with MluI. The resulting 288 bp internal *orbS* fragment released from each amplicon was used to replace the corresponding fragment in pBBR1MCS-orbS. Due to loss of a 12 bp MluI fragment in the *orbS* gene contained on pBBR1MCS-orbS during replacement of the 288 bp MluI fragment, the 725 bp HindIII-PstI fragment of both alanine substitution plasmids (containing *P_orbS_* and the first 516 bp of the *orbS* coding sequence) was replaced by the corresponding fragment from pBBR1MCS-orbS. The ‘mega-primer’ variation of overlap extension-PCR (51,52) was used to generate pBBR1MCS-OrbS-C203A, pBBR1MCS-OrbS-C209A and pBBR1MCS-OrbS-CtetraA. Here, mutagenic forward primers Cysala3for, Cysala4for or cysalaallfor were used in combination with orbSrev2 to generate the megaprimers that contain the desired codon substitution(s). These were used as a reverse primer in combination with orbSfor in a second PCR to generate the full length allele, which was then ligated to pBBR1MCS following digestion with HindIII and BamHI. These alleles were transferred to pET14b for protein expression by amplification with orbsfor7 and orbSrev2 and ligation to the vector NdeI and BamHI sites.

Plasmids expressing C-terminal truncated OrbS derivatives were generated by amplifying *orbS* with primer orbSfor in combination with each of the reverse primers orbSΔCrev1 to orbSΔCrev7 and ligating the products to pBBR1MCS following restriction of the plasmid and amplicons with HindIII and BamHI. Plasmids expressing *orbS* alleles independently of the Fur-regulated P_orbS_ promoter were constructed by amplifying the *orbS* and *orbS-CtetraA* alleles using primers orbS-fwd-HindIII and orbS-rev-BamHI. Each amplicon was then ligated between the HindIII and BamHI sites of pBBR1MCS-2 to generate pBBR2-orbS_ΔP_ and pBBR2-orbS-CtetraA_ΔP_. The orbS-fwd-HindIII primer included a translation termination codon in the 5′ tail to ensure termination of translation of vector-encoded *lacZα* mRNA, thereby preventing formation of a LacZ-OrbS fusion protein. In these plasmids, *orbS* retains its native Shine-Dalgarno sequence and is transcribed from the *aphA2* (Km^R^) and *lacZ* promoters present on the pBBR1MCS-2 vector.

pBBR1MCS-fur::Tp was constructed by insertion of the BamHI Tp^R^ cassette from p34E-Tp into the BglII site located within the *B. cenocepacia fur* gene carried by pCAL4. The 2.64 kb *fur*::Tp SalI fragment was then transferred to the SalI site of the allelic replacement plasmid pSHAFT2 to generate pSHAFT2-fur::Tp. To construct pSHAFT2-Δfur::Tp, pSHAFT2-fur::Tp was digested with SmaI, which cuts at four sites within the plasmid (within the vector *cat* (Cm^R^) gene, both sides of the Tp^R^ cassette, and between *fur* and the downstream ureidoglycolate lyase (*allA*) gene), to generate four DNA fragments. These products were self-ligated and introduced into *E. coli* CC118 (λpir) with selection for chloramphenicol- and trimethoprim-resistant transformants. Colonies were screened for plasmids in which the SmaI fragment containing the region of *fur* located between the site of insertion of the Tp^R^ cassette and the stop codon had not been reincorporated into the reconstructed plasmid. The *fur* complementation plasmid, pBBR2-fur3, was constructed in two steps by first deleting 0.89 kb DNA between the SmaI sites present on pCAL4 to generate pBBR1-fur2 (this manipulation removes all *B. cenocepacia* DNA carried on pCAL4 that is present downstream of *fur*) followed by transfer of the 1.13 kb SacI-SmaI *fur* DNA fragment to the corresponding sites of pBBR1MCS-2. The pSNUFF-pchE′::Km allelic replacement plasmid was constructed by amplifying a 1.4 kb DNA fragment from the *B. cenocepacia* H111 genome encompassing the 3′ terminal 35 bp of *pchR*, the *pchR*-*pchE* intergenic region, and 1.245 kb from the 5′ end of *pchE* with primers pchEfor and pchE_Rv2_NheI, digesting the PCR product with NheI and KpnI, and ligating it to the XbaI and KpnI sites of pSNUFF to generate pSNUFF-pchE′. The Km^R^ cassette was then removed from p34E-Km by digestion with SmaI and blunt end ligated into the ZraI site within the *pchE′* fragment present in pSNUFF-pchE′. pSHAFT2 derivatives were maintained in *E. coli* CC118 (λpir).

pSNUFF and pSNUFF3Cm are pEX18Tp-*pheS*-derived allelic replacement vectors for introducing unmarked mutations into the *B. cenocepacia* genome (53; H.L.Spiewak and M.S.T., unpublished results). pSNUFF-ΔorbS was constructed by SOE-PCR (54) whereby a 379 bp DNA fragment containing the first 18 codons of *B. cenocepacia orbS* together with 325 bp of upstream DNA was amplified with primers orbSmut1 and orbSmut2, and fused to a 412 bp DNA fragment containing the last 23 codons of *orbS* along with 343 bp of DNA located downstream of *orbS* that was amplified with primers orbSmut3 and orbSmut4. The splicing of the two amplicons involved a second PCR with primers orbSmut1 and orbSmut4, and occurred through overlapping complementarity between the two amplicons. The spliced PCR product was digested with BamHI and HindIII, ligated to the corresponding sites within pSNUFF, and introduced into *E. coli* JM83 for blue/white colony screening. To construct pSNUFF3Cm-orbS-CtetraA, the *orbS*::CtetraA allele was amplified from pBBR1MCS-*orbS*::CtetraA with primers orbSfor6 and CtetraA_Rv. Separately, a 267 bp region located downstream of *orbS* was amplified with primers orb500_fwd and orbSmut4 using *B. cenocepacia* H111 DNA as the template. Both PCR products contained a 21 bp region of complementarity located downstream of *orbS* which allowed for their splicing in a subsequent SOE PCR reaction using primers orbSfor6 and orbSmut4. The spliced amplicon was then cloned between the BamHI and HindIII sites of pSNUFF3Cm.

pRLG770-P_orbH_ was constructed by annealing complementary oligonucleotides orbHds6_oligofwd and orbHds6_oligoRv, that include nucleotides at positions -37 to + 5 of the *orbH* promoter (i.e. corresponding to P_orbHds6_; Supplementary Figure S1B), and then ligating the resulting double-stranded oligonucleotide to the EcoRI and HindIII sites of pRLG770.

All PCR amplifications for DNA cloning utilised proofreading thermostable DNA polymerases and cloned DNA inserts were confirmed by DNA sequencing.

### Construction of *B. cenocepacia* mutants

To generate the Δ*orbS* mutant, pSNUFF-ΔorbS was introduced into the *E. coli* conjugal donor strain SM10 (λpir) to facilitate subsequent delivery of the plasmid into *B. cenocepacia* H111. Merodiploid strains resulting from integration of the plasmid into the *B. cenocepacia* genome by homologous recombination were selected on M9 salts agar containing trimethoprim. Following PCR confirmation of genomic integration of the plasmid at the *orbS* locus, recombinants were grown overnight in LB without selection and serial dilutions were spread plated onto M9 agar containing 0.1% (w/v) chlorophenylalanine to select for recombinants that had lost the integrated plasmid. Chlorophenylalanine-resistant, trimethoprim-sensitive colonies were PCR screened for the presence of the Δ*orbS* allele, which has lost 180 codons from the 220 codon *orbS* coding sequence, with primers orbS_check_fwd and orbS_check_rv. To replace the chromosomal *orbS* gene with the *orbS*-CtetraA allele, pSNUFF3Cm-ΔorbS-CtetraA was introduced into H111ΔorbS by conjugation and merodiploids resulting from recombinational integration of the plasmid into the *B. cenocepacia* chromosome were identified by PCR following selection of exconjugants on M9-CAA agar containing 25 μg/ml trimethoprim and 10 μg/ml tetracycline. Plasmid pDAI-SceI, which encodes the yeast I-SceI meganuclease, was introduced into merodiploid strains. This enzyme causes a DNA break at the I-SceI target site located within the vector backbone of the chromosomally integrated pSNUFF3Cm plasmid, and thereby induces recombinogenic DNA repair, resulting in loss of the integrated plasmid. Colonies that grew on Lennox agar containing 125 μg/ml tetracycline and 100 μg/ml ampicillin, but not on IST agar containing 25 μg/ml trimethoprim, were subjected to PCR with primers orbsfor7 and orbsrev2 and the amplified DNA digested with BglI. This enzyme only cuts the amplicon if it is derived from the wild-type *orbS* gene but not if it is derived from a recombinant harbouring the *orbS*-CtetraA allele. Amplicons that were resistant to BglI digestion were sequenced to confirm replacement of all four cysteine codons by alanine codons.

To construct the *B. cenocepacia fur*::Tp mutant, *E. coli* SM10 (λpir) was used to introduce pSHAFT2-fur::Tp into strain 715j, and *B. cenocepacia* exconjugants were selected on M9 agar containing trimethoprim. Colonies were then patched onto LB agar containing chloramphenicol to identify candidate double crossover recombinants in which the wild-type *fur* gene had been replaced by the *fur*::Tp allele. Chloramphenicol-sensitive colonies were verified as *bona fide fur* mutants by PCR screening using primers FuroutFor and FuroutRev. *B. cenocepacia* Δ*fur*::Tp mutants were constructed by delivering pSHAFT2-Δfur::Tp into H111 and its derivatives by conjugation with SM10 (λpir) followed by selection for trimethoprim-resistant *B. cenocepacia* exconjugants on M9-CAA agar. Colonies that were chloramphenicol sensitive were PCR screened with primers furcheck_fwd and furcheck_Rv to detect colonies with a disrupted *fur* gene. To inactivate *pchE*, pSNUFF-*pchE*::Km was conjugated into *B. cenocepacia* H111 and derivatives thereof. *B. cenocepacia* ex-conjugants were selected on Lennox agar containing kanamycin. Colonies that were kanamycin-resistant but trimethoprim-sensitive were PCR screened with primers pchE_for2 and pchE_Rv2 to confirm that the chromosomal *pchE* gene had been disrupted by insertion of the *aphA2* (Km^R^) cassette (4.5 kb product for the *pchE*::Km allele compared to 3.2 kb for WT *pchE*).

### Protein expression and purification

*E. coli* BL21 (DE3) containing pET14b-derived plasmids expressing OrbS and OrbS-CtetraA were grown in 500 ml of autoinduction medium (6 g/l Na_2_HPO_4_, 3 g/l KH_2_PO_4_, 20 g/l tryptone, 5 g/l yeast extract, 5 g/l NaCl, 0.5% (v/v) glycerol, 0.05% (w/v) glucose, 0.2% (w/v) lactose) at 37°C on an orbital shaker (250 rpm) until cells reached late log phase (OD_600_ 0.8–1.0). Cultures were then grown for a further 16 h at 22°C with aeration whereupon the cells were harvested by centrifugation. BugBuster Protein Extraction Reagent (Millipore) containing lysozyme (1 mg/ml) was used to lyse cell pellets at 5 ml/g of cell pellet. OrbS proteins were found in the insoluble protein fraction which was separated from the soluble fraction by centrifugation at 16,000 x *g* for 20 min. OrbS proteins were resolubilised overnight at 4°C on a rotating platform by adding 25 ml TGD buffer (50 mM Tris-HCl (pH 7.9), 10% (v/v) glycerol, 1 mM DTT, 50 mM NaCl) supplemented with 0.25% Sarkosyl. Following this treatment, the remaining insoluble material was separated by a further centrifugation step as described above. To further purify OrbS proteins, the solubilised protein was subjected to nickel affinity purification using a HisTrap column (GE Life Sciences) pre-equilibrated with binding buffer (50 mM Tris-HCl (pH 7.5), 500 mM NaCl, 10% (v/v) glycerol, 1 mM DTT, 20 mM imidazole, 0.25% Sarkosyl). After applying the protein sample (in TGD buffer but with imidazole added to 20 mM) and washing the column with binding buffer, OrbS was eluted in a buffer containing 50 mM Tris-HCl (pH 7.5), 500 mM NaCl, 10% (v/v) glycerol, 1 mM DTT, 500 mM imidazole and 0.25% Sarkosyl. Purified proteins were refolded by dialysis with two changes of HGD buffer (50 mM HEPES (pH 7.4), 5% (v/v) glycerol, 1 mM DTT, 50 mM NaCl) before a final dialysis step into storage buffer (50 mM HEPES (pH 7.9), 50% (v/v) glycerol, 50 mM NaCl, 100 μM DTT, 100 μM EDTA). Protein purity was monitored by electrophoresis in 12% SDS-polyacrylamide gels.

His-tagged *E. coli* σ^70^ was purified by growing BL21(DE3) containing pOPINF-sigma70 at 37°C in 6 litres LB until the culture reached OD_600_ ∼0.6, whereupon the culture was equilibrated at 16°C followed by addition of isopropyl β-D-1-thiogalactopyranoside (IPTG) to a final concentration of 0.5 mM. Following overnight incubation, the bacterial cells were harvested by centrifugation and suspended in lysis buffer A (20 mM Tris, pH 8.0, 250 mM NaCl, 1 mM tris(2-carboxyethyl)phosphine (TCEP), 10% glycerol, 20 mM imidazole). Cells were lysed by sonication and the lysate cleared by centrifugation (18,000 rpm for 40 min at 4°C) prior to loading onto a nickel affinity column pre-equilibrated with lysis buffer A. His-tagged σ^70^ was eluted with a linear 20–500 mM gradient of imidazole in buffer A. The eluted σ^70^ protein was pooled and dialysed against heparin buffer A (20 mM Tris, pH 8.0, 75 mM NaCl, 1 mM TECP, 10% glycerol) overnight. The sample was then loaded onto a heparin column and the protein was eluted with a linear 75–800 mM gradient of NaCl in heparin buffer A. The eluted σ^70^ protein was then concentrated and loaded onto a size exclusion column (HiLoad 16/600 Superdex 200 pg) pre-equilibrated with a buffer containing 25 mM HEPES pH 7.5, 250 mM NaCl, 10% glycerol, 1 mM TCEP. The purified σ^70^ protein, which was homogeneous as judged by SDS-PAGE, was concentrated to 15.8 mg/ml and stored at -80°C before use.

### Analysis of siderophore production

For agar plate assays, overnight cultures grown in EB medium were standardised to an OD_600_ of 1.0 in a 1 ml volume. Cells were then harvested by centrifugation and resuspended in PBS. Either 1 μl or 20 μl of culture was then spot plated onto CAS agar plates containing either 10 μM or 60 μM FeCl_3_. For measurement of siderophore production in broth cultures, the procedure of Kvitko *et al.* (55) was followed. Bacterial strains were grown overnight in EB medium supplemented with 50 μM FeCl_3_. The next day the OD_600_ was determined and cells harvested. 900 μl of culture supernatant that had been passed through a 0.22 μm filter was then mixed with 100 μl of CAS reagent (0.605 mg/ml CAS, 0.729 mg/ml HDTMA, 100 μM FeCl_3_). After incubation for 30 min at room temperature the A_630_ was measured and the value subtracted from that of the reference (900 μl EB medium plus 100 μl CAS containing 50 μM FeCl_3_) to obtain the ΔA_630_ value which was then normalised to the OD_600_.

### β-Galactosidase assays

Cells harbouring pKAGd4 in which the *P_orbH_* or *P_orbS_* promoter was located upstream of the *lacZ* gene were grown in M9-CAA or LB media supplemented with chloramphenicol and either the metal ion chelator 2,2′-dipyridyl or iron (III) chloride. Where bacterial cultures also contained *orbS* expression plasmids, the medium was supplemented with an additional selective antibiotic as appropriate. When the cultures had reached an OD_600_ of ∼0.2 to 0.4, cells were chilled on ice for at least 20 min, following which 25–100 μl of the cell suspension was added to Z buffer (final volume 1ml) at 30°C, and the cells permeabilised with chloroform-sodium dodecyl sulphate. Following equilibration to 30°C (15 min) the assay was initiated with *o*-nitrophenyl-β-D-galactopyranoside (ONPG) as the substrate (56). The assays were performed in duplicate (technical replicates) on three independent cultures for each plasmid construct or condition (n = 3). Values presented (in Miller units) were background corrected by subtraction of the activity measured in cells harbouring the parental plasmid pKAGd4 (and either pBBR1MCS-2 or pBBR1MCS-5, where appropriate), grown under identical conditions.

### Real-time quantitative polymerase chain reaction (qPCR)

*B. cenocepacia* overnight cultures were diluted into EB medium supplemented with 50 μM FeCl_3_ or 100 μM 2,2′-dipyridyl and grown to OD_600_ = 0.5. RNA was extracted using the Macherey-Nagel Nucleospin RNA kit according to the manufacturer's instructions and the concentration determined by Nanodrop (Thermo Scientific) and standardised to ∼50 ng/μl. A High-Capacity cDNA Reverse Transcription Kit (Applied Biosystems) was used to synthesise complementary DNA (cDNA) according to the manufacturer's instructions. cDNA was then used as a template in 40 cycle qPCR assays that were performed according to the manufacturer's instructions, using 2x qPCR SYBR green Master Mix (Primer Design Precision) and primers orbI_int_fwd and orbI_int_Rv (to measure *orbI* expression) or qPCRrpoD_F and qPCRrpoD_Rv (as an internal housekeeping gene expression control) using the 7900HT AbiPrism Sequence Detection System. Reactions in which reverse transcriptase or cDNA were omitted served as negative controls. Relative *orbI* expression was calculated using the 2^–ΔΔCT^ method with *rpoD* transcripts used as an internal referencing control.

### *In vitro* transcription assays

Plasmid pRLG770-P_orbH_ served as the template for *in vitro* transcription assays. The presence of the tandem *rrnB* transcription terminators located downstream from the cloning site in the vector gives rise to a discrete ‘*orbH’* transcript of 156 nucleotides. pRLG770 also contains the σ^70^-dependent RNA I promoter, P1, which results in generation of a 108 bp transcript. Multiple-round transcription reactions were performed as described previously (57) with *E. coli* RNAP holoenzyme (NEB) or *E. coli* RNAP core enzyme (NEB) reconstituted with σ^OrbS^, σ^OrbS-CtetraA^, σ^OrbS-C196A^, σ^OrbS-C199A^, σ^OrbS-C203A^ or σ^OrbS-C209A^. Reconstitution was performed in 1 x RNAP reaction buffer (40 mM Tris-HCl (pH 7.5), 150 mM KCl, 10 mM MgCl_2_, 1 mM dithiothreitol, 0.01% Triton X-100™, provided by the manufacturer) at 30°C for 10 min. Reactions were then initiated at the same temperature with NTPs and template DNA for 5 min using *E. coli* holoenzyme or 30 min for reconstituted polymerase. Each 25 μl reaction mixture contained 1 unit of RNAP, 1 x reaction buffer, 150 ng supercoiled template DNA, 200 μM ATP, 200 μM GTP, 200 μM CTP, 10 μM UTP, 5 μCi (0.185 MBq) of [α-^32^P] UTP (800 Ci/mmol; Perkin-Elmer) and 25 μM Fe(NH_4_)_2_(SO_4_)_2_, or FeCl_3_, where appropriate. Reactions were terminated with 25 μl of stop solution (95% formamide, 20 mM EDTA, 0.05% bromophenol blue). Samples, containing equal amounts of template DNA, were fractionated in a 5.5% acrylamide gel containing 7 M urea, and transcripts were visualised using a FujiFilm FLA3000 phosphorimager.

### Electrophoretic shift mobility assays (EMSAs)

The 415 bp *P_orbH_* probe (-348 to +67) was released from plasmid pBS-porbH with BamHI and HindIII, following which 15 μl of probe (30 ng/μl) was labelled at the HindIII end with 2 μCi of [α-^32^P]dATP (3000 Ci/mmol; Perkin-Elmer) using DNA polymerase I Klenow fragment at room temperature for 30 min. Following labelling, DNA was purified using the GeneJet PCR purification kit (ThermoFisher Scientific) as per the manufacturer's instructions. A 10 μl reaction mix was then set up using 1 unit of *E. coli* core RNAP (NEB), 1 μl of 10 x buffer (400 mM Tris acetate (pH 7.9), 400 mM KCl, 10 mM DTT, 10 mM MgCl_2_), 1 μg of sigma factor and where appropriate 25 μM of transition metal salts. Reconstitution of RNAP was carried out at 37°C for 10 min, followed by the addition of labelled DNA probe and incubation continued for a further 30 min. Heparin, at a final concentration of 200 μg/ml, was then added to the binding complex for a further 5 min. 10 μl samples were then loaded under tension onto a pre-run 4.5% polyacrylamide gel and electrophoresed for 80 min at 100 V in 0.5 x TBE buffer. Band shifts were visualised using a FujiFilm FLA3000 phosphorimager.

### Biolayer interferometry

Assays were performed in RNAP buffer (40 mM Tris-HCl (pH 7.5), 150 mM KCl, 10 mM MgCl_2_, 1 mM DTT, 0.01% (v/v) Triton X-100) with freshly made solutions of Fe(II) as required. Ni-NTA Biosensors (fortéBIO, Pall Corp., USA) were hydrated in RNAP buffer for a minimum of 10 min before use. Each assay was performed according to the steps in Supplementary Table S4. For each interaction, six assays were performed with a range of concentrations of core RNAP from 62.5 nM to 2000 nM diluted in RNAP buffer. Data were analysed using the Data Analysis v9.0 software (fortéBIO, Pall Corp., USA). The dissociation rates (*k*_d_ (s^–1^)), association rates (*k*_a_ (M^–1^ s^–1^)) and the binding affinity constants (*K*_D_ (M)) were calculated using a global analysis of the six data runs across six concentrations of core RNAP from the start of association to the end of dissociation.

### Computational resources

Amino acid sequence alignments were made using Clustal Omega (https://www.ebi.ac.uk/Tools/msa/clustalo/).

### Statistical analyses

The number of experiments carried out and the statistical analyses applied to the data are indicated in the text. Statistical tests were performed using GraphPad Prism. Biolayer interferometry data were processed using the Octet Data Analysis Software (fortéBIO) which performs non-linear regression curve fitting of the data with standard errors for the calculated parameters.

## RESULTS

### Fur regulation of *orbS* expression is insufficient to fully account for increased ornibactin synthesis under iron-depleted conditions

**Figure 1.**
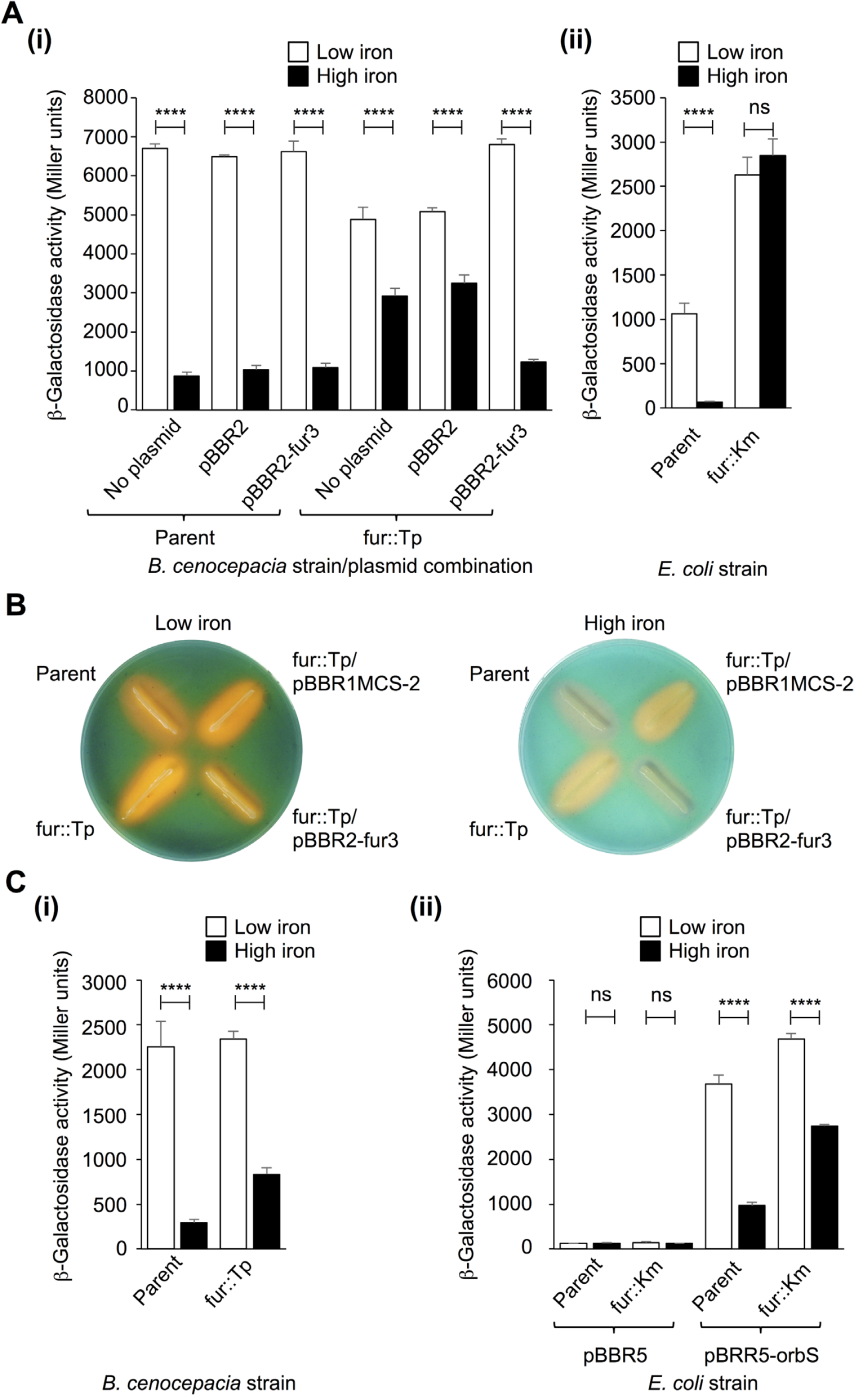
Fur regulation of *orbS* alone cannot account for iron-responsive synthesis of ornibactin. (**A**). Effect of inactivation of *fur* on iron-regulation of *P_orbS_*. (i) *B. cenocepacia* 715j (parent) or 715jfur::Tp (fur::Tp) containing pKAGd4-P_orbS-69_ with or without pBBR1MCS-2 (pBBR2) or pBBR2-fur3; (ii) *E. coli* QC771 (parent) or the isogenic *fur* mutant, QC1732 (fur::Km), containing pKAGd4-P_orbS_. (**B**). Effect of inactivation of *fur* on siderophore production by *B. cenocepacia*. Representative images of *B. cenocepacia* 715j (parent) and 715jfur::Tp (fur::Tp) streaked on CAS plates to show siderophore production. Left, standard CAS plate (low iron; 10 μM FeCl_3_); right, high iron CAS plate (60 μM FeCl_3_). Yellow-orange halos show the presence of ornibactin while pale purple halos are the result of pyochelin production. (**C**). Effect of inactivation of *fur* on iron-regulation of *P_orbH_*. (i) *B. cenocepacia* 715j (parent) or 715jfur::Tp (fur::Tp) containing pKAGd4-P_orbHds6_; (ii) *E. coli* QC771 (parent) or the isogenic *fur* mutant, QC1732 (fur::Km), containing pKAGd4-P_orbHds6_ and either pBBR1MCS-5 (pBBR5) or pBBR5-orbS. In (**A**) and (**C**), β-galactosidase activity measurements were performed on cultures grown in LB containing chloramphenicol together with either 2,2′-dipyridyl, to achieve iron deficient conditions (white bars), or ferric chloride, for iron replete conditions (black bars). Kanamycin was also included for strains harbouring pBBR1MCS-2 derivatives and gentamicin for pBBR1MCS-5 derivatives. Activities shown were obtained following subtraction of the activity of the same strain containing pKAGd4 assayed under the same conditions (background control). Statistical significance was tested using two-way ANOVA with a Tukeys’ post-test (n = 3), **** = *P*< 0.0001, ns = not significant.

Experiments using a reconstituted system in *E. coli* indicated that the iron-responsive regulator, Fur, directly represses transcription from the *orbS* promoter (*P_orbS_*) under conditions of iron sufficiency (29). Accordingly, expression of a *P_orbS_-lacZ* fusion (Figure 1Ai) and ornibactin production (Figure 1B) were increased in the *B. cenocepacia fur* insertion mutant, 715jfur::Tp, under iron-replete conditions, compared to the parent strain and the complemented mutant. However, it was noticed that for *B. cenocepacia* cells growing under both iron-replete and iron-depleted conditions, inactivation of Fur did not increase *orbS* expression to a level greater than or equal to that achieved by the parent strain growing under iron-depleted conditions (Figure 1Ai), as would have been expected, and which was the case for the *E. coli fur* mutant (Figure 1Aii). These observations suggested the possibility that Fur, directly or indirectly, acts as an activator of *orbS* expression in addition to its role as a direct repressor, in *B. cenocepacia*, but only acts as a repressor of *orbS* in the heterologous *E. coli* host. It is noted that the dysregulation of iron homeostasis brought about by deletion of *fur* might exert pleiotropic effects that confound interpretation of these experiments (e.g. changes in *P_orbS_-lacZ* fusion plasmid copy number). Nevertheless, as *orbS* expression appeared to be fully derepressed in the *E. coli fur* mutant we measured transcription from the OrbS-dependent *orbH* promoter (*P_orbH_*) in the *B. cenocepacia fur* mutant, and in an *E. coli fur* mutant expressing *orbS*, under iron-replete and iron-starved conditions. As expected, cultures of both the *B. cenocepacia fur* mutant and the OrbS^+^*E. coli fur* mutant carrying a *P_orbH_-lacZ* fusion had higher β-galactosidase activities during growth under iron-replete conditions than those of the parent strains, due to the absence of Fur-mediated repression of *orbS* in the mutant strains (Figure 1C). Strikingly, further significant increases in *orbH* expression were observed for iron-depleted cultures. The latter suggests that Fur regulation of *P_orbS_* cannot fully account for increased ornibactin production under conditions of iron-starvation – in the reconstituted *E. coli* experiments *orbS* is fully derepressed in the *fur* mutant and yet *orbH* expression was still responsive to iron availability; *orbH* expression in *B. cenocepacia* was also iron-responsive independently of Fur, but in addition to Fur-mediated repression, expression of *orbS* is possibly subject to further regulation.

### OrbS contains a C-terminal iron-responsive domain

**Figure 2.**
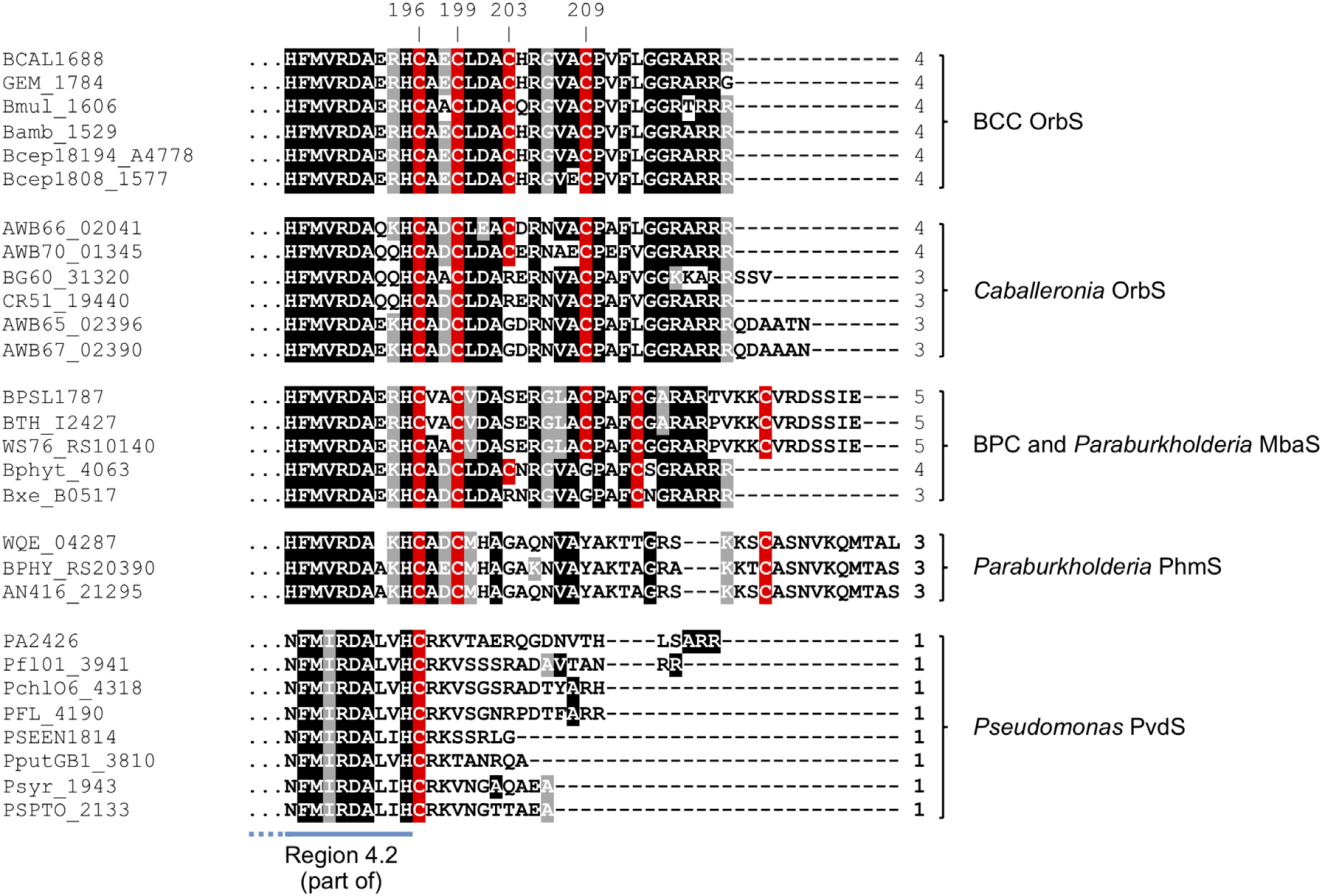
Comparison of the C-terminal amino acid sequences of OrbS, MbaS and PhmS orthologues with the well-characterized *P. aeruginosa* IS ECF σ factor, PvdS, and its orthologues. The sequence of residues 186–220 of *B. cenocepacia* OrbS (BCAL1688) is shown aligned with the corresponding region of representative OrbS, MbaS, PhmS and PvdS orthologues from other species (BCC, *B. cepacia* complex; BPC, *B. pseudomallei* complex), including *P. aeruginosa* PvdS (PA2426). The region shown includes ∼10 residues of region 4.2 (indicated by the blue bar). Assignment of the sigma factor as OrbS in members of the genus *Caballeronia* is based on bioinformatics analysis of the OrbI and OrbJ NRPS orthologues which are predicted to generate ornibactin (results not shown). For other assignations, see Butt and Thomas (25). Amino acids that are identical at the corresponding position in ≥ 50% of sequences are shown in white font with black highlighting, whereas amino acids that are similar are shown with grey highlighting. Cysteine residues are shown in white font with red highlighting. The total number of C-terminal cysteine residues in each σ factor is shown on the right of the corresponding sequence. The positions of the four cysteine residues of *B. cenocepacia* OrbS that are referred to in this study are indicated above the alignment. Note: *Caballeronia* and *Paraburkholderia* are recently described genera that contain former members of *Burkholderia sensu lato* (104,105).

The realization that Fur-mediated regulation of OrbS abundance could not fully account for regulation of ornibactin production prompted us to consider the possibility that OrbS itself is post-translationally regulated by iron. OrbS is considered to be an ECF σ factor and these have been classified into 157 phylogenetic groups based on their structure, regulatory mechanism and genomic context (58). Members of 19 groups possess C-terminal extensions of 50–200 amino acids (58). The presence of C-terminal extensions generally correlates with the apparent absence of a cognate anti-σ factor and hence these C-terminal regions are likely to have a regulatory function (58–62). OrbS has been assigned to the ECF243 group and is not regulated by an anti-σ factor/σ factor regulator (29,58). Therefore, we compared the C-terminal regions of OrbS proteins from members of the *Burkholderia* genus that possess an ornibactin gene cluster with PvdS orthologues (also ECF243 group members) from *Pseudomonas* spp., which are activated by anti-σ factor degradation (42,44), to identify sequences that might constitute an OrbS autoregulatory domain. This analysis revealed that OrbS is only 5 amino acids longer at its C-terminus than the archetypal PvdS from *P. aeruginosa*, and is at most 17 amino acids longer than the shortest PvdS orthologue, from *P. entomophila* (Figure 2). However, whereas the amino acid sequences of region 4.2 in both types of σ factor are conserved, consistent with the very similar -35 sequences present at OrbS- and PvdS-dependent promoters (29,37), the amino acid sequences located C-terminal to region 4.2 diverge (Figure 2). Particularly noteworthy is the presence of a cluster of four cysteine residues in the C-terminal region of OrbS (C196, C199, C203 and C209), one of which (C196) is located at the boundary with region 4.2 and is conserved in OrbS and PvdS orthologues, while the three more distally located cysteine residues are unique to OrbS (Figure 2). Interestingly, MbaS and PhmS, which regulate expression of genes specifying biosynthesis and utilisation of the ornibactin-related siderophores, malleobactin and phymabactin, in species closely related to *B. cenocepacia*, were also observed to contain cysteine-rich C-terminal regions (25; Figure 2).

**Figure 3.**
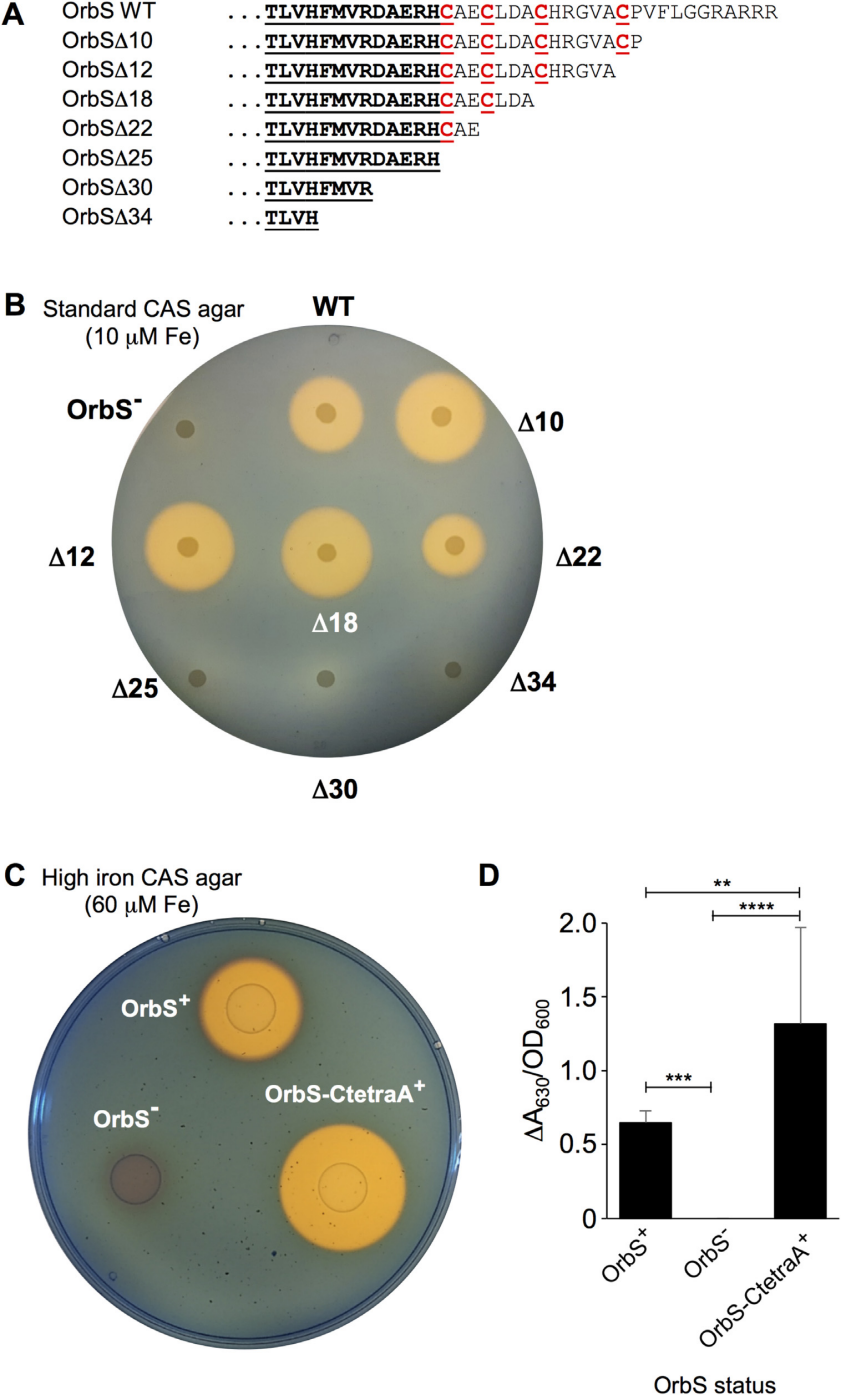
The C-terminal cysteine residues of OrbS function to inhibit ornibactin production in the presence of iron. (**A**). C-terminal sequences of truncated OrbS derivatives. Amino acid residues 183–220 of the wild-type (WT) sequence are shown, with cysteine residues indicated in red font and underlined. Residues that form part of region 4.2 are also underlined. (**B**). Ornibactin production by *B. cenocepacia* OM3 (Pch^–^*orbS*::mini-Tn*5*Tp) expressing C-terminally truncated OrbS derivatives. Cultures of OM3 containing pBBR1MCS-based plasmids encoding OrbS and its truncated derivatives were spotted on standard CAS agar (10 μM FeCl_3_) and incubated overnight (a representative plate is shown; see Supplementary Figure S2 for statistical analysis). The production of ornibactin is evidenced by a blue to orange colour change around the growth following overnight incubation. (**C**). Cultures of *B. cenocepacia* H111Δ*fur* Δ*pchE* harbouring different chromosomal *orbS* alleles giving rise to the indicated phenotypes, were grown overnight in EB medium at 37°C, normalised and spot plated onto high-iron CAS agar (60 μM FeCl_3_). (**D**). Quantitation of siderophore production. Supernatants from overnight cultures grown in EB medium containing 50 μM FeCl_3_, were mixed with CAS reagent and a colour change was detected spectrophotometrically by comparing the A_630_ to a reference containing CAS reagent and culture broth. The normalised data presented is the average of three independent experiments. Error bars show standard deviation. Statistical significance was tested using two-way ANOVA with a Tukeys’ post-test (n = 3), **** = *P*< 0.0001, *** = *P*< 0.001, ** = *P*< 0.01.

To investigate the function of the C-terminal region of OrbS, we examined the effect of expressing truncated OrbS proteins on production of ornibactin. Plasmid-encoded C-terminal truncated OrbS derivatives (Figure 3A) were expressed in the *B. cenocepacia orbS* null mutant, OM3, which as well as failing to produce ornibactin, does not produce the alternative siderophore, pyochelin. The latter characteristic allows the restoration of ornibactin production by complementation with *orbS* to be readily visualised using a CAS agar assay (29). Deletion of up to 18 amino acids from the C-terminus of OrbS (OrbSΔ10, Δ12 and Δ18) did not exert a detrimental effect on its ability to restore ornibactin production; in fact these truncations appeared to enhance ornibactin synthesis (Figure 3B). Deletion of a further four amino acids from the C-terminus (OrbSΔ22) resulted in a marked decrease in ornibactin production, while removal of another 3–12 amino acids (OrbSΔ25, Δ30 and Δ34) abolished production of the siderophore (Figure 3B). These data suggest that amino acids located C-terminal to region 4.2 are not essential for OrbS activity and ornibactin synthesis, whereas deletions that encroach into region 4.2 (C196 marks the boundary of PvdS region 4.2 helix-turn-helix; (63)) impair holoRNAP binding at the -35 elements of OrbS-dependent promoters, thereby abolishing ornibactin production.

Although the region of OrbS located C-terminal to region 4.2 was not essential for OrbS activity, it was noted that deletion of up to 18 amino acids from the extension resulted in increased ornibactin production, suggesting that this region might be important in regulating OrbS activity (Figure 3B). In preliminary experiments to investigate this possibility, a plasmid, pBBR1MCS-OrbS-CtetraA, coding for an OrbS variant in which all four cysteine residues were replaced by alanine, was shown to be able to complement ornibactin production by the OM3 strain on standard CAS agar plates (Supplementary Figure S3). Therefore, to better assess the function of the OrbS cysteine-rich C-terminal region in ornibactin production, we constructed *B. cenocepacia* H111 *Δfur ΔpchE* mutant strains harbouring chromosomal *orbS* WT, null or CtetraA alleles. In addition to the absence of regulation by Fur, the Δ*fur* Δ*pchE* mutant does not produce the *B. cenocepacia* secondary siderophore, pyochelin, thereby eliminating any pyochelin-mediated effect on the cellular iron pool and simplifying quantification of ornibactin production on CAS agar plates (64–66). The *B. cenocepacia* H111 *ΔorbS Δfur ΔpchE* mutant did not produce ornibactin on CAS agar plates containing a high concentration (60 μM) of iron (*ΔorbS* in Figure 3C). In the *orbS*^+^ *ΔfurΔpchE* strain the orange-yellow halo surrounding the bacterial growth indicated that ornibactin was produced (*orbS*^+^ in Figure 3C). However, when the chromosomal copy of *orbS* was replaced by the *orbS-CtetraA* allele, ornibactin production was increased compared to the wild type allele, similar to observations made with the OrbS Δ10, Δ12, and Δ18 variants.

To estimate the relative amounts of ornibactin produced by the wild type and *orbS-CtetraA* strains, broth cultures were grown under iron-replete conditions and a liquid CAS based assay was performed to detect ornibactin in the culture supernatants. The results showed that the *orbS-CtetraA Δfur ΔpchE* strain produced approximately twice as much ornibactin as the parent *Δfur ΔpchE* strain (Figure 3D). As expected, ornibactin was not detected in culture supernatants obtained from the *ΔorbS Δfur ΔpchE* strain. Taken together these results suggest that in the presence of iron the C-terminal cysteine residues of OrbS function to inhibit ornibactin production.

### The C-terminal cysteine cluster of OrbS is required for iron-responsive transcription regulation of the *orbI* promoter

Measurement of ornibactin production by cultures grown on solid and in liquid media suggested that the C-terminal cysteine cluster of OrbS functions to inhibit OrbS activity in the presence of iron (Figure 3). To investigate the ability of OrbS and OrbS-CtetraA to activate expression of an OrbS-dependent promoter (*P_orbI_*) under iron-replete and iron-depleted conditions, qPCR was used to quantify *orbI* mRNA in the *B. cenocepacia orbS-CtetraA Δfur ΔpchE* strain relative to the *Δfur ΔpchE* strain (OrbS^+^). Expression of *orbI* in *B. cenocepacia* growing under low iron conditions was similar for *Δfur ΔpchE* cells expressing either the wild type OrbS protein or OrbS-CtetraA (relative expression level ∼1). However, for cultures grown under high iron conditions there was ∼5-fold more *orbI* transcript detected when OrbS-CtetraA was expressed (Supplementary Figure S4). These data again suggest that the C-terminal cysteine cluster of OrbS functions to regulate OrbS transcriptional activity in response to iron, i.e. OrbS activity is inhibited in the presence of iron.

### The C-terminal cysteine residues of OrbS are required for iron-mediated inhibition of transcription *in vitro*

**Figure 4.**
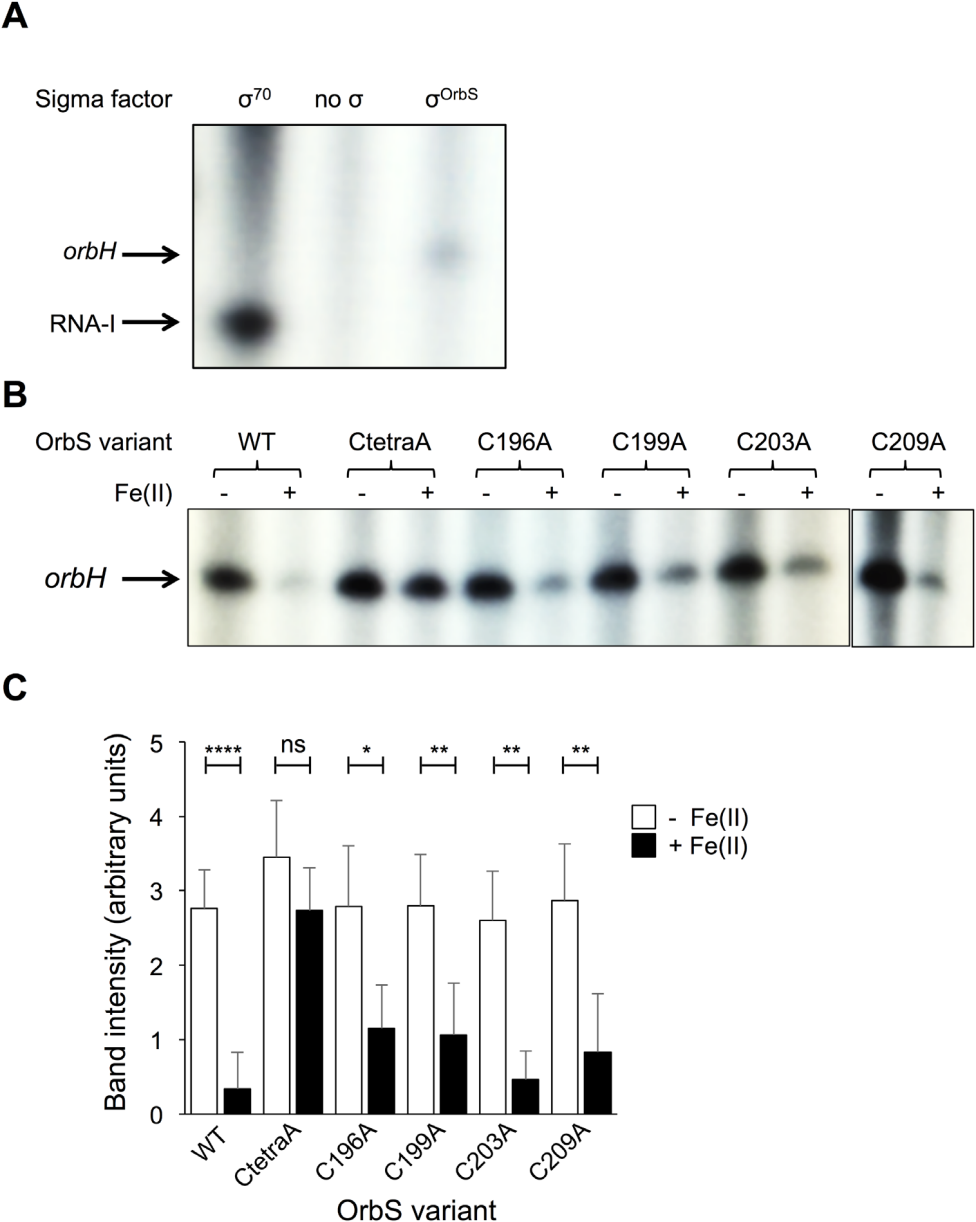
OrbS is a σ factor that is inhibited by ferrous iron. (**A**). Dependency of the *orbH* promoter (P*_orbH_*) on OrbS. *E. coli* core RNAP, σ^70^-RNAP and OrbS-RNAP holoenzymes were tested for their ability to initiate transcription from the *P_orbH_* promoter. The RNA I promoter in the vector (pRLG770) acted as a positive control for transcription by σ^70^-RNAP. RNA transcripts were separated by electrophoresis in a 5.5% denaturing polyacrylamide gel and transcripts were visualised using a phosphorimager. (**B**). Effect of Fe(II) on OrbS-dependent transcription. OrbS and the indicated cysteine-substituted OrbS variants were incubated with core RNAP in the presence or absence of 25 μM Fe(NH_4_)_2_(SO_4_)_2_ before addition of *P_orbH_* template DNA and NTPs to initiate transcription from the promoter. RNA transcripts were separated and visualised as described in (**A**). The image is a representative of three independent experiments. (**C**). Transcript intensities quantified from the experiments described in (**B**) using ImageJ software (no iron addition, white bars; Fe(II) addition, black bars). Error bars show standard deviation. * = *P*< 0.05, ** = *P*< 0.01, **** = *P*< 0.0001, ns = not significant, following Two-way ANOVA, Sidak's multiple comparison test. Significance is shown for the indicated pairs of metal-treated and untreated OrbS variants.

The previous identification of OrbS as an ECF σ factor was based on its high degree of overall similarity to the functionally characterised ECF σ factor, PvdS, and its requirement for transcription from ‘*orb’* gene promoters (29,37). Therefore, before investigating whether and how iron regulates OrbS activity, we needed to establish that OrbS actually functions as a σ factor by associating with core RNAP and specifically directing transcription from an OrbS-dependent promoter (*P_orbH_*) *in vitro*. Previous work, and results presented herein, showed that expression of *orbS* permitted transcription from OrbS-dependent promoters in *E. coli* (Figure 1Aii; 37), and so if OrbS was acting as a σ factor we should be able to reconstitute *E. coli* core RNAP with either purified *E. coli* σ^70^ or with OrbS for *in vitro* transcription assays. These assays used a pRLG770 derivative, pRLG770-P_orbH_, which contained *P_orbH_* and the σ^70^-dependent promoter, P1, as the template. The latter directs synthesis of the replication inhibitor RNA, RNA I (67–69). As expected, in the presence of σ^70^ holoRNAP only the RNA I transcript was generated from pRLG770-P_orbH_ (Figure 4A). In contrast, OrbS-reconstituted RNAP holoenzyme (σ^OrbS^ holoRNAP) gave rise to a specific ‘*orbH*’ transcript of the expected size, but not the RNA I transcript. This showed that *in vitro* transcription from *P_orbH_* requires association of OrbS with core RNAP, confirming that OrbS functions as a σ factor.

To determine whether iron exerts a direct effect on OrbS activity, OrbS-dependent transcription from the *P_orbH_* promoter was analysed in the presence of Fe(II) *in vitro*. The results showed that Fe(II) exerted a strong inhibitory effect on OrbS-dependent transcription (Figure 4B and C). Importantly, the inhibitory effect of Fe(II) on OrbS activity was abolished when all four C-terminal cysteine residues were substituted. Individual substitutions of C196, C199 or C209 also led to a decrease in the sensitivity of OrbS to Fe(II), although not to the same extent as substitution of all four cysteine residues; substitution of C203 exerted little effect on the sensitivity of OrbS to Fe(II) (Figure 4B and C).

### Fe(II) inhibits formation of an open complex at *P_orbH_*

Electrophoretic mobility shift assays (EMSAs) were carried out in the presence of heparin at 37°C, where shifting of promoter-bearing DNA fragments by holoRNAP would be expected to occur only following formation of the more stable open complex (70,71). When either *E. coli* core RNAP or *E. coli* σ^70^ holoRNAP were mixed with a radiolabelled *P_orbH_* DNA fragment, the mobility of the DNA was the same as that observed in the absence of protein (Supplementary Figure S5). In contrast, when σ^OrbS^ holoRNAP was incubated with the *P_orbH_* DNA fragment, a retarded complex was observed, confirming that recognition of *P_orbH_* requires OrbS (Supplementary Figure S5). However, when Fe(II) was included in the EMSA binding buffer, formation of the *P_orbH_*:σ^OrbS^ holoRNAP complex was impaired. In contrast, in the presence of σ^OrbS-CtetraA^ holoRNAP, the fraction of the *P_orbH_* probe that was retarded was similar in the presence or absence of Fe(II) (Supplementary Figure S5). These results suggest that inhibition of OrbS-dependent transcription by Fe(II) (Figure 4) is likely to occur prior to formation of the promoter open complex.

### Fe(II) inhibits interaction of OrbS with core RNAP

To determine whether Fe(II) inhibits the assembly of OrbS with core RNAP or a subsequent step in the transcription initiation cycle, we investigated the interaction between immobilized OrbS and core *E. coli* RNAP using biolayer interferometry (BLItz) in the presence and absence of Fe(II) (Supplementary Figure S6). BLItz is used to measure the kinetics and affinity of molecular interactions in solution by analysis of time-resolved changes in the interference patterns of white light reflected from the surface of a loaded probe upon binding of an analyte. Therefore, BLItz provides additional quantitative information for protein interactions compared to other methods, such as cross-linking and pull-down assays. Ni-NTA probes were loaded with His-tagged OrbS, OrbS-CtetraA, or *E. coli* σ^70^ and exposed to *E. coli* core RNAP (the analyte) in the absence or presence of Fe(II). Any binding of core RNAP to the immobilized sigma factor would increase the number of protein molecules bound to the probe resulting in a shift in the interference pattern that can be measured in real time. The data were fitted to association/dissociation curves to calculate binding and dissociation constants. Binding of *E. coli* core RNAP to *E. coli* σ^70^ was essentially unaffected by the addition of Fe(II); i.e. similar association (*k*_a_), dissociation (*k*_d_) and equilibrium dissociation (*K*_D_) constants were obtained in the absence and presence of Fe(II) (Table 1). The *k*_d_ and *K*_D_ values for *E. coli* core RNAP binding to *E. coli* σ^70^ obtained from the BLItz experiments were similar to those calculated from Surface Plasmon Resonance data also obtained using Ni-NTA immobilised His-tagged σ^70^ (72). Thus, these *E. coli* data suggest that BLItz experiments should be suitable for investigating the effect of Fe(II) on the OrbS:core RNAP interaction. The OrbS:core RNAP interaction parameters measured in the absence of Fe(II) were similar to those determined for σ^70^; however, in the presence of Fe(II) we were unable to fit the OrbS:core RNAP association phase (*k*_a_) within acceptable error limits, suggesting that Fe(II) inhibits OrbS binding to core RNAP (Table 1). This was not the case for the OrbS-CtetraA:RNAP interaction, where similar *k*_a_ values were obtained in the absence and presence of Fe(II), and although the calculated *k*_d_ value was greater than that obtained in the absence of Fe(II), it was similar to that obtained for *E. coli* σ^70^ in the presence of Fe(II) (Table 1). These data suggest that the failure to determine an association rate constant for the OrbS:core RNAP interaction in the presence of Fe(II) was unlikely to result from Fe(II) acting directly on core RNAP. Therefore, it is likely that Fe(II)-mediated inhibition of OrbS-dependent transcription and binding at *P_orbH_* results from impaired interaction between OrbS and core RNAP mediated by a mechanism involving the C-terminal cysteine cluster of OrbS.

**Table 1.** Effect of Fe(II) on binding of *E. coli* core RNAP to *E. coli* σ^70^, *B. cenocepacia* OrbS and OrbS-CtetraA

	Absence of Fe(II)			Presence of Fe(II)		
Parameter	σ^70^	OrbS	OrbS-CtetraA	σ^70^	OrbS	OrbS-CtetraA
*k*_a_ (M^–1^ s^–1^)	5.5 × 10^4^ (± 1.5 × 10^3^)	5.4 × 10^3^ (± 1.8 × 10^2^)	1.5 x 10^4^ (± 3.9 × 10^2^)	6.1 × 10^4^ (± 2.2 × 10^3^)	6.7 × 10^0^ (± 1.2 × 10^3^)	9.1 × 10^3^ (± 3.7 × 10^2^)
*k*_d_ (s^–1^)	1.9 × 10^–3^ (± 5.1 × 10^–5^)	4.5 × 10^–4^ (± 3.9 × 10^–5^)	3.0 × 10^–4^ (± 2.9 × 10^–5^)	2.5 × 10^–3^ (± 8.3 × 10^–5^)	2.4 × 10^–4^ (± 1.0 × 10^–4^)	1.5 × 10^–3^ (± 1.4 × 10^–4^)
*K*_D_ (M)	3.5 × 10^–8^ (± 8.0 × 10^–9^)	8.3 × 10^–8^ (± 2.9 × 10^–8^)	2.0 × 10^–8^ (± 7.0 × 10^–9^)	4.1 × 10^–8^ (± 1.1 × 10^–8^)	3.6 × 10^–5^ (± 4.8 × 10^–4^)	1.6 × 10^–7^ (± 6.0 × 10^–8^)

## DISCUSSION

Restricting the availability of the essential trace element, iron, is one of several ‘nutritional immunity’ strategies used by hosts to control infections by bacterial pathogens (73–77). Unsurprisingly these bacteria have evolved elaborate mechanisms to scavenge iron, including the production of siderophores (78,79). Bacteria that produce siderophores express receptors for the loaded siderophore to facilitate iron uptake (24,79). Since siderophores are ‘public goods’, controlling the expression of genes necessary for their synthesis and utilisation is important for both individual bacteria and the wider population, and is thus subject to regulation (40,80). A common component of this regulation is repression of iron acquisition genes by the global regulator Fur under conditions of iron sufficiency (81–84). Previously, it was concluded that Fur-mediated repression of *orbS* transcription was the main mechanism by which production of the siderophore ornibactin is regulated in *B. cenocepacia* and that a signal transduction mechanism involving a σ factor regulator (e.g. an anti-σ factor) is not involved (29). Here we reveal that OrbS activity is in fact subject to post-translational regulation by iron through a C-terminal cysteine cluster, such that intracellular Fe(II) impairs OrbS binding to core RNAP and consequently inhibits OrbS-dependent ornibactin production, uptake and utilisation.

Of the more than 15 ECF σ groups that lack cognate anti-σ factors but possess C-terminal extensions, members of three such groups have been investigated (58,62). The most similar to OrbS are the metal ion-regulated σ factors CorE and CorE2 that were formerly placed in the ECF44 group that is now incorporated into the ECF238 group (58,85–87). CorE is activated by binding Cu(II) at its cysteine-rich (Cys_(184)_-X_4_-Cys-X_2_-Cys-X_1_-Cys) C-terminal domain, permitting productive interaction with core RNAP to regulate genes involved in copper homeostasis (85). The cysteine-rich (Cys_(173)_-Cys-X_3_-Cys-X_2_-Cys-X_1_-Cys) C-terminal domain of CorE2 is essential for activation by Cd(II) and Zn(II), and the transcription of genes involved in resistance to these metals, with metal specificity being determined by Cys_174_, which is absent from CorE (86). The OrbS cysteine-rich motif (Cys_(196)_-X_2_-Cys-X_3_-Cys-X_5_-Cys) differs from those of CorE and CorE2. This motif presumably contributes to the selection of Fe(II) as the cognate metal ion for OrbS and the resulting inhibition of OrbS activity, rather than the metal ion-dependent activation observed for CorE and CorE2. However, the precise mechanisms by which the interaction of these different metal ions with the cysteine-rich C-terminal regions of CorE, CorE2 and OrbS results in activation or inactivation of these ECF σ factors are still not clear. Nevertheless, it is possible to make some informed suggestions based upon our current understanding of related ECF σ factors.

The simplest model to explain the inhibitory action of Fe(II) on OrbS activity is to posit that Fe(II) chelation induces a conformation change in the σ factor C-terminal region that prevents its interaction with the -35 core promoter element, either through distortion of the σ_4_ domain or occlusion of its DNA-binding surface. As a mode of σ factor ‘inactivation’ this suggestion has a precedent in the mechanism of action of the *B. subtilis* anti-σ factor, RsiW, which blocks the interaction of the σ_4_ domain of SigW with the -35 region of its target promoter (88). However, our BLItz analysis suggests that Fe(II) inhibits OrbS interaction with core RNAP, a mode of action that appears to be more commonly utilised by anti-σ factors (89). Although most of the binding surfaces involved in the interaction of σ^70^ family members with core RNAP involve the σ_2_ domain, the σ_4_ domain does make contacts with the flap tip helix of the β subunit and the β′ zinc finger domain (90), and it is possible that disruption of these interactions due to iron-dependent reorganisation of the OrbS C-terminus destabilises the σ-core interaction sufficiently to impair formation of the OrbS-RNAP holoenzyme. However, there is an alternative possibility. We note that OrbS, and OrbS-like σ factors such as MbaS and PhmS, also harbour N-terminal extensions which are absent in the related σ factor PvdS of the pseudomonads (Supplementary Figure S7). Perhaps a long-range interaction between the N- and C-termini of OrbS could be facilitated by conformational changes that occur at the C-terminus upon binding of Fe(II)? Such an interaction may be permissible due to the flexibility of the σ_2_-σ_4_ interdomain linker and may lock OrbS into an alternative conformation that prevents its interaction with core RNAP.

The proposal that ligand mediated conformational changes in the C-terminal region alters the ability of OrbS to interact with core RNAP has similarities to the regulation of some members of the ECF41 and ECF42 groups. The long C-terminal extension of ECF41 σ factors folds into a SnoaL-like domain which is likely to regulate the activity of the fused σ factor in response to the binding of a low molecular weight ligand (91). In the absence of ligand the SnoaL-like domain maintains the fused σ factor in a closed (inhibitory) conformation through interaction between the C-terminal region of the SnoaL-like domain and the σ_2_-σ_4_ interdomain linker (62,92). Thus, like CorE and CorE2, ligand binding would activate ECF41 σ factors, possibly by promoting the adoption of a more open conformation that permits productive interaction with core RNAP. ECF42 σ factors possess a long C-terminal extension that ends with a tetratricopeptide repeat (TPR) domain (93). TPR domains are implicated in protein-protein interactions (94). Interaction between the σ_4_ proximal region of the C-terminal extension and the second and third α-helices of the σ_4_ domain is required for ECF42 σ activity (92,95). It has been suggested that binding of a ligand (possibly another protein) to the TPR domain results in conformational changes in the σ_4_ proximal region of the C-terminal extension to suppress ECF42 σ factor activity, presumably by disrupting the interaction with the second and third α-helices of the σ_4_ domain (92). Thus, as for OrbS, ligand binding to ECF42 σ factors inhibits their activity. However, it is not yet clear whether ligand binding alters the ability of ECF42 σ factors to interact with core RNAP or to impair DNA recognition, or both. The proposal that Fe(II) binding at the C-terminal region of OrbS impairs binding to core RNAP, as suggested here, offers the advantage that molecules of RNAP are not taken out of commission by their recruitment to inactivated σ factors.

**Figure 5.**
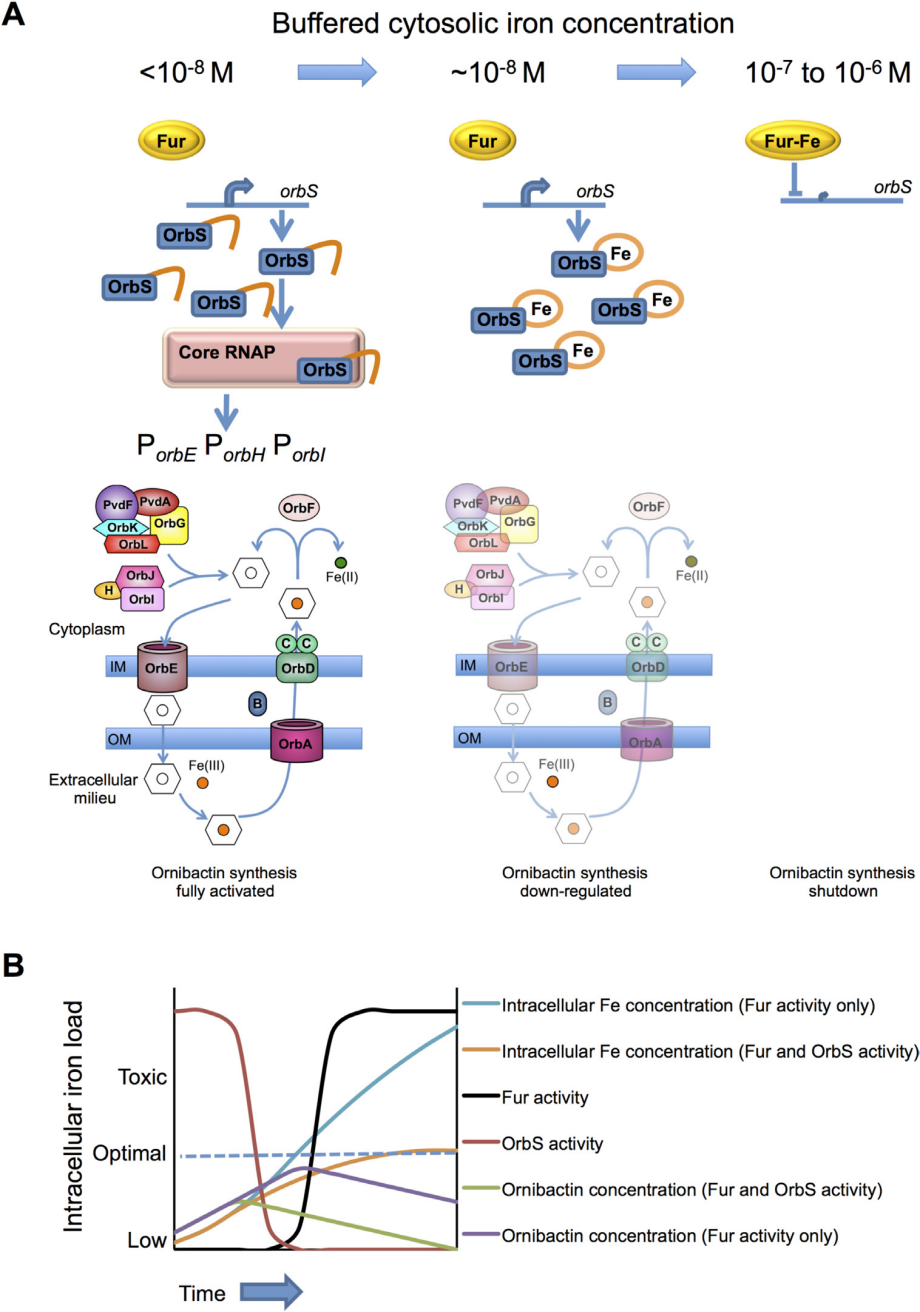
Proposed model to account for dual-level regulation of OrbS activity in response to changes in cytoplasmic iron concentration. (**A**). Under conditions of iron starvation (buffered cytosolic Fe(II) concentration < 10^–8^ M) the Fur repressible *orbS* gene is expressed. Apo-OrbS binds core RNAP and genes for ornibactin synthesis and utilisation are expressed from the *orbE*, *orbH* and *orbI* operon promoters (P*_orbE_*, P*_orbH_*, P*_orbI_*). Ornibactin (open hexagon) is secreted across the inner membrane (IM) by OrbE and released into the extracellular milieu where it binds Fe(III) (orange circle) with high affinity. Fe(III)-loaded ornibactin binds to its outer membrane receptor (OrbA) and is translocated to the cytoplasm by the OrbBCD transporter. In the cytosol the OrbF protein reductively releases Fe(II) (green circle) from Fe(III)-loaded ornibactin. As the intracellular iron pools are replenished (buffered cytosolic Fe(II) concentration ∼10^–8^ M), the cysteine-rich C-terminal region of OrbS (amber squiggle) binds Fe(II) and OrbS no longer interacts with core RNAP. Consequently OrbS-dependent genes are switched off and synthesis and uptake of Fe(III)-loaded ornibactin is progressively diminished due to protein turnover and an increasing bacterial population. Nevertheless, this diminishing amount of capacity (indicated by the faded synthesis and utilisation pathway) continues to supply iron. When the buffered cytosolic Fe(II) concentration reaches ∼10^–7^–10^–6^ M, the Fur-Fe(II) complex forms, repressing Fur-regulated genes, including *orbS*, and ornibactin production is shutdown. (**B**). Graphical model of iron accumulation. Consider a cell under conditions of iron starvation, both Fur and OrbS are in their apo-forms; Fur-regulated genes, including *orbS* are derepressed and apo-OrbS is active. Assume that in a given time this allows the production of 10 ornibactin molecules and that in the same time period 3 of these 10 are lost from the system through degradation etc. It is also assumed that each ornibactin molecule provides 2 Fe atoms for the cell as a consequence of siderophore recycling. The numbers are not intended to represent measured values but are chosen to illustrate synthesis, degradation and recycling of ornibactin. In this model, we assume that OrbS has a higher affinity for iron than Fur and hence its activity (red) is inhibited and ornibactin (green) production is switched off before the cell is fully replete with iron. The continuing ion scavenging of extant ornibactin results in the iron content of the cell rising to a plateau below the toxicity threshold (orange). If this OrbS regulation is removed from the system, such that ornibactin production is solely regulated by Fur (black), then excess ornibactin (purple) is produced resulting prolonged accumulation of iron to potentially toxic levels (blue).

The realisation that OrbS is subject to post-translational regulation raises the question of why cytoplasmic iron is the signal, rather than iron-loaded ornibactin, as might be expected for a canonical CSS-type mechanism. A possible explanation might be that CSS mechanisms allow some bacteria to exploit xenosiderophores, as well as endogenous siderophores. Another question is why a two-level (Fur and OrbS) regulatory circuit in which two transcription regulators act in response to the same signal, cytoplasmic Fe(II), has evolved to control ornibactin production, rather than control being exerted exclusively through Fur? Fur is a global regulator controlling expression of multiple genes in many bacteria (96–99). Data obtained for *Salmonella* Fur protein indicated that under conditions in which the buffered cytosolic Fe(II) concentration decreased by about an order of magnitude (to ∼10^–8^ M), the fractional occupation by Fur of a DNA fragment containing a Fur-binding site decreased to < 10% (100). Thus, Fur-mediated control is tuned to respond close to the optimum intracellular Fe(II) concentration. It could be argued that if ornibactin synthesis was exclusively controlled by Fur, then ornibactin synthesis and utilisation genes would likely be expressed when the buffered cytosolic Fe(II) concentration is ≤ 10^–8^ M and not switched off until it is > 10^–7^ M. However, the ornibactin synthesis and utilisation proteins as well as ornibactin itself are relatively long-lived and would continue to supply iron to the cells. Hence, regulation exclusively tuned to respond close to optimal buffered intracellular Fe(II) concentrations is likely to result in the uptake of excess iron, perhaps to toxic levels (36) as ornibactin continues to supply iron after transcription of the ‘ornibactin’ genes has been switched off (Figure 5). By introducing a second level of regulation, such potentially dangerous overshoots in the intracellular Fe(II) pool could be avoided. If the affinity of OrbS for Fe(II) is greater than that of Fur, as ornibactin-mediated iron uptake rebuilds the intracellular iron pools, the higher affinity of OrbS for Fe(II) would allow the cell to switch off expression of ornibactin synthesis genes below the optimal intracellular Fe(II) concentration, thereby ‘anticipating’ the continuation of Fe(II) supply from extant ornibactin. Such a regulatory strategy, which switches off expression of OrbS-dependent genes once intracellular iron concentration is on an upward trend, minimizes expenditure of resources producing excess public goods that could only benefit competitors. Hence, a future priority should be to investigate the thermodynamics of OrbS and Fe(II) interactions to establish how the binding affinity compares with that of Fur.

The inability of OrbS-CtetraA to respond to iron does not imply that the coordination of iron is solely dependent on the OrbS C-terminal cysteine cluster, because there are conserved aspartate and histidine residues within this region (Figure 2), and given the inherent flexibility of proteins, these could act as metal-binding ligands in combination with different subsets of the cysteine residues. Further mutational analyses will be needed to determine which OrbS residues are essential for iron-binding. Moreover, one of the shortcomings of our current understanding of OrbS is the lack of information on the nature and properties of the iron-cofactor. This knowledge gap results from the limited availability of pure OrbS and the relative instability of the purified protein upon storage. To overcome this, further investment of resources directed towards increasing the yields of pure OrbS protein and optimizing storage conditions to facilitate the application of a wider range of biochemical and biophysical techniques to characterize the OrbS metal centre will be required. Here it has been shown that inhibition of OrbS activity *in vitro* occurred simply by addition of Fe(II) under reducing conditions, and did not require provision of an additional source of sulfide, as might be expected for the assembly of an iron-sulfur cluster. This observation suggests that OrbS could be inactivated by acquisition of a mononuclear iron-centre located at its cysteine-rich C-terminal region. However, in early work it was possible to switch the *E. coli* [4Fe-4S] FNR protein between active and inactive states by addition and removal of Fe(II) under reducing conditions (101,102). It was later shown that during disassembly of the FNR [4Fe-4S] cluster, sulfide is retained by the protein and can be utilized to reconstruct the cluster when Fe(II) is provided under reducing conditions (103). It is possible that a similar process permits assembly of an iron-sulfur cluster that inactivates OrbS. Accurate mass measurements of OrbS under oxidizing and reducing conditions should establish whether apo-OrbS possesses any sulfur adducts (persulfide formation) and thereby determine whether an FNR-like cluster repair mechanism could operate with OrbS. Once improved OrbS purification and storage protocols are in place, further biochemical and biophysical characterization should allow the details of the interplay between cytoplasmic Fe(II) availability and the relative affinities of Fur and OrbS for Fe(II) to be determined, thereby leading to the formulation of a detailed mechanism for optimal ornibactin synthesis.

## DATA AVAILABILITY

Source data are provided as supplementary files.

## Supplementary Material

gkac137_Supplemental_File

## Notes

Present address: Aaron T. Butt, Manchester Metropolitan University, John Dalton Building, Chester Street, Manchester M1 5GD, UK.

Present address: Christopher D. Banyard. IN-PART Publishing Ltd., Parkhead House, Carver Street, Sheffield S1 4FS, UK.

Present address: Kirsty Agnoli, Dept. of Microbiology, Institute of Plant Biology, University of Zürich, Zollikerstrasse 107, CH-8008 Zürich, Switzerland.

Present address: Muslim I. Mohsin, Department of Pathological Analyses, Faculty of Science, University of Kufa, Kufa, Najaf, Iraq.

Present address: Rebecca Lomax, Department of Materials Science and Engineering, Kroto Research Institute, University of Sheffield, Sheffield, UK.
