# Supplementary material for: The Burkholderia cenocepacia iron starvation σ factor, OrbS, possesses an on-board iron sensor
Source: Nucleic Acids Res. 2022 Mar 2;50(7):3709–26. doi: 10.1093/nar/gkac137 (PMC9023288; doi:10.1093/nar/gkac137)
Supplement: gkac137_Supplemental_File [file gkac137_supplemental_file.docx]

**Supplementary Data**

**Table S1. Bacterial strains**

| **Strain name** | **Genotype or Description** | **Source or reference** |
| --- | --- | --- |
| ***B. cenocepacia* strains**  715j  715jfur::Tp  KLF1  OM3  H111  H111ΔorbS  H111orbS-CtetraA  H111Δfur  H111ΔorbS Δfur  H111orbS-CtetraA Δfur  H111ΔpchE  H111ΔorbS ΔpchE  H111orbS-CtetraA ΔpchE  H111Δfur ΔpchE  H111ΔorbS Δfur ΔpchE  H111orbS-CtetraA Δfur ΔpchE | CF isolate, prototroph (Orb^+^ Pch^+^)  715j containing a Tp^R^ cassette insertion in the *fur* gene (Orb^+^ Pch^+^)  Spontaneous pyochelin-negative mutant of 715j (Orb^+^ Pch^-^)  KLF1 harbouring a mini-Tn*5*Tp insertion in the *orbS* gene (Orb^-^ Pch^-^)  CF isolate, prototroph (Orb^+^ Pch^+^)  H111 containing a deletion of 180 codons within *orbS* (Orb^-^ Pch^+^)  H111 in which the cysteine codons at positions 196, 199, 203 and 209 of the chromosomal *orbS* gene have been substituted by alanine codons  H111 in which the chromosomal *fur* gene has been replaced by a Δfur::Tp^R^  allele  H111Δ*orbS* containing the Δ*fur*::Tp^R^ allele  H111orbS-CtetraA containing the Δ*fur*::Tp^R^ allele  H111 containing a Km^R^ cassette inserted in the *pchE* gene (Orb^+^ Pch^-^)  H111ΔorbS containing the *pchE*::Km^R^ allele  H111orbS-CtetraA containing the *pchE*::Km^R^ allele  H111Δ*fur* containing the *pchE*::Km^R^ allele  H111ΔorbS containing the Δ*fur*::Tp^R^ and *pchE*::Km^R^ alleles  H111orbS-CtetraA containing the Δ*fur*::Tp^R^ and *pchE*::Km^R^ alleles | (1,2)  This study  (3)  (3)  (4)  This study  This study  This study  This study  This study  This study  This study  This study  This study  This study  This study |
| ***E. coli* strains**  JM83  MC1061  S17-1 (λpir)  SM10 (λpir)  CC118 (λpir)  BL21(DE3)  QC771 (GC4468)  QC1732 | F^-^ *ara* (*lac-proAB*) *rpsL* ϕ80d*lacZM15* (Sm^R^)  F^–^ *araD139* Δ*(ara, leu)7697* Δ*(lacIPOZYA)X74 galK16 galE15 rpsL150 mcrA0 mcrB1 hsdR2 relA::IS2 spoT e14^-^* λ*^-^* (Sm^R^)  *thi pro-82* (Δ*(frsA-ykfC)) hsdR recA glvB*::*RP4*-*2*-*tet*::Mu-1 *kan*::Tn*7* integrant λ*pir* (Tp^R^ Sm^R^)  *thi thr leu tonA lacY supE recA mhpC*::RP4-2-Tc::Mu λpir (Km^R^)  Δ(*ara-leu*) *araD* Δ*lacX74 galE galK phoA20 thi-1 rpsE rpoB argE*_(am)_ *recA1* λ*pir* (Rf^R^)  F^–^ *ompT* *gal* *dcm* *lon* *hsdS_B_*(*r_B_*^–^*m_B_*^–^) λ(DE3 [*lacI* *lacUV5*-*T7 gene 1* *ind1* *S_am_7* *nin5*])  F^-^ Δ(*argF-lac*)U169 *rpsL* (Sm^R^)  QC771 Δ*fur*::*kan* (Km^R^ Sm^R^) | (5)  (6)  (7,8,9)  (7,8)  (10)  (11)  (12)  (13) |

Abbreviations: Km^R^, resistant to kanamycin; Rf^R^, resistant to rifampicin; Sm^R^, resistant to streptomycin; Tp^R^, resistant to trimethoprim; Orb, ornibactin phenotype; Pch, pyochelin phenotype

**Table S2. Plasmids**

| **Plasmid name** | **Description** | **Source or reference** |
| --- | --- | --- |
| pKAGd4  pKAGd4-P_orbHds6_  pKAGd4-P_orbHds96_  pKAGd4-P_orbS_  pKAGd4-P_orbS-69_  pBBR1MCS  pBBR1MCS-orbS  pBBR1MCS-orbSΔ10  pBBR1MCS-orbSΔ12  pBBR1MCS-orbSΔ18  pBBR1MCS-orbSΔ22  pBBR1MCS-orbSΔ25  pBBR1MCS-orbSΔ30  pBBR1MCS-orbSΔ34  pBBR1MCS-orbS-CtetraA  pBBR1MCS-orbS-C196A  pBBR1MCS-orbS-C199A  pBBR1MCS-orbS-C203A  pBBR1MCS-orbS-C209A  pCAL4  pBBR1MCS-fur::Tp  pBBR1-fur2  pBBR1MCS-2  pBBR2-fur3  pBBR2-orbS_ΔP_  pBBR2-orbS-CtetraA_ΔP_  pBBR1MCS-5  pBBR5-orbS  pET14b  pET14b-orbS  pET14b-orbS-CtetraA  pET14b-orbS-C196A  pET14b-orbS-C199A  pET14b-orbS-C203A  pET14b-orbS-C209A  pRLG770  pRLG770-P_orbH_  pBS-porbH  pSHAFT2  pSHAFT2-fur::Tp  pSHAFT2-Δfur::Tp  pSNUFF  pSNUFF3Cm  pSNUFF-ΔorbS  pSNUFF3Cm-ΔorbS-CtetraA  pSNUFF-pchE’  pSNUFF-pchE’::Km  p34E-Tp  p34E-Km  pDAI-SceI  pOPINF  pOPINF-sigma70 | Mobilisable BHR transcriptional reporter plasmid derived from pPR9TT (Cm^R^ Ap^R^)  pKAGd4 containing 42 bp *P_orbH_* fragment (-37 to +5) fused to *lacZ*  pKAGd4 containing 51 bp *P_orbH_* fragment (-43 to +8) fused to *lacZ*  pKAGd4 containing *P_orbS_* (-178 to +181 relative the transcription start site) fused to *lacZ*  pKAGd4 containing *P_orbS_* (-69 to +181 relative the transcription start site) fused to *lacZ*  Mobilisable BHR cloning vector; IncP and ColE1 compatible (Cm^R^)  pBBR1MCS containing the *B. cenocepacia* *orbS* gene and its native promoter  pBBR1MCS-orbS lacking 10 3’-terminal codons of *orbS*  pBBR1MCS-orbS lacking 12 3’-terminal codons of *orbS*  pBBR1MCS-orbS lacking 18 3’-terminal codons of *orbS*  pBBR1MCS-orbS lacking 22 3’-terminal codons of *orbS*  pBBR1MCS-orbS lacking 25 3’-terminal codons of *orbS*  pBBR1MCS-orbS lacking 30 3’-terminal codons of *orbS*  pBBR1MCS-orbS lacking 34 3’-terminal codons of *orbS*  pBBR1MCS containing the *orbS-CtetraA* allele and its native promoter  pBBR1MCS containing the *orbS-C196A* allele and its native promoter  pBBR1MCS containing the *orbS-C199A* allele and its native promoter  pBBR1MCS containing the *orbS-C203A* allele and its native promoter  pBBR1MCS containing the *orbS-C209A* allele and its native promoter  pBBR1MCS containing *B. cenocepacia fur* gene on a 2.0 kb SalI fragment  pCAL4 with BamHI Tp^R^ cassette from p34E-Tp inserted into BglII site of *fur*  pCAL4 from which *B. cenocepacia* genomic DNA downstream of *fur* has been deleted  Mobilisable BHR cloning vector; IncP and ColE1 compatible (Km^R^)  pBBR1MCS-2 containing the *B. cenocepacia fur* gene and its native promoter  pBBR1MCS-2 containing the *B. cenocepacia* *orbS* gene without its native promoter  pBBR1MCS-2 containing the *orbS-CtetraA* allele without its native promoter  Mobilisable BHR cloning vector; IncP and ColE1 compatible (Gm^R^)  pBBR1MCS-5 containing the *B. cenocepacia* *orbS* gene and its native promoter  High level expression of N-terminally 6xHis-tagged proteins (Ap^R^)  pET14b containing the *orbS* gene  pET14b containing the *orbS-CtetraA* allele  pET14b containing the *orbS* gene with a cys to ala codon substitution at codon 196  pET14b containing the *orbS* gene with a cys to ala codon substitution at codon 199  pET14b containing the *orbS* gene with a cys to ala codon substitution at codon 203  pET14b containing the *orbS* gene with a cys to ala codon substitution at codon 209  Derivative of pKM2 for *in vitro* transcription assays (Ap^R^)  pRLG770 containing the *P_orbHds6_* promoter (−37 to +5)  pBluescript II KS containing 415 bp *orbH* promoter fragment. Source of *P_orbH_* for EMSAs  Allelic replacement vector for constructing marked (cassette insertion) mutants.  R6K origin of replication, RP4 origin of transfer (*oriT*) (Ap^R^ Cm^R^)  pSHAFT2 containing 2.65 kb SalI *fur*::Tp fragment from pBBR1MCS-fur::Tp  pSHAFT2-fur::Tp containing a deletion of the 3’-terminal 119 codons of *fur*  Allelic replacement vector for constructing unmarked (i.e. deletion) mutants (Tp^R^)  Allelic replacement vector for constructing unmarked (i.e. deletion) mutants (Tp^R^, Cm^R^)  pSNUFF containing the Δ*orbS* allele  pSNUFF3Cm containing the *orbS-CtetraA* allele  pSNUFF containing 1.25 kb from the 5’ end of *B. cenocepacia pchE*  pSNUFF-pchE’ containing the Km^R^ cassette from p34E-Km inserted within *pchE*’  Source of *dfrB2* (Tp^R^) cassette (Ap^R^, Tp^R^)  Source of *aphA2* (Km^R^) cassette (Ap^R^, Km^R^)  Source of I-SceI nuclease for stimulating allelic replacement by pSNUFF3Cm derivatives (Tc^R^)  High level expression of N-terminally 6xHis-tagged proteins (Ap^R^)  pOPINF containing the *E. coli rpoD* gene encoding σ^70^ inserted between the KpnI and HindIII sites | (3)  (14)  This study  (3)  (3)  (15)  (3)  This study  This study  This study  This study  This study  This study  This study  This study  This study  This study  This study  This study  (16)  This study  This study  (17)  This study  This study  This study  (17)  This study  Novagen  This study  This study  This study  This study  This study  This study  (18)  This study  (3)  (19)  This study  This study  (20)  H. Spiewak and MST, unpublished  This study  This study  This study  This study  (21)  (19)  (22)  (23)  This study |

Abbreviations: Ap^R^, specifies ampicillin resistance; Cm^R^, specifies chloramphenicol resistance; Gm^R^, specifies gentamicin resistance; Tp^R^, specifies trimethoprim resistance; Km^R^, specifies kanamycin resistance; BHR, broad host-range

**Table S3. Primers and complementary oligonucleotide pairs for cloning**

| **Primer name** | **Primer sequence (5´→3´)** | **Description or purpose** |
| --- | --- | --- |
| orbSfor | GCGCAAGCTTCGGTTCGTCAGGAACATGAA | Forward primer for amplification of *orbS*. HindIII site. |
| orbSrev2 | GCGCGGATCCGGCTTGCGTCATGATCGGAAA | Reverse primer for amplification of *orbS*. BamHI site. |
| orbSΔCrev1 | GCGCGGATCCTTACGGACAGGCGACGCCGCGAT | Construction of pBBR1MCS-orbSΔ10 |
| orbSΔCrev2 | GCGCGGATCCTTAGGCGACGCCGCGATGGCACG | Construction of pBBR1MCS-orbSΔ12 |
| orbSΔCrev3 | GCGCGGATCCTTACGCGTCGAGGCATTCCGCGC | Construction of pBBR1MCS-orbSΔ18 |
| orbSΔCrev4 | GCGCGGATCCTTATTCCGCGCAGTGGCGCTCCG | Construction of pBBR1MCS-orbSΔ22 |
| orbSΔCrev5 | GCGCGGATCCTTAGTGGCGCTCCGCGTCGCGCA | Construction of pBBR1MCS-orbSΔ25 |
| orbSΔCrev6 | GCGCGGATCCTTAGCGCACCATGAAATGCACGA | Construction of pBBR1MCS-orbSΔ30 |
| orbSΔCrev7 | GCGCGGATCCTTAATGCACGAGCGTCTGCGACA | Construction of pBBR1MCS-orbSΔ34 |
| Cysala1rev | GCGCCACGCGTCGAGGCATTCCGCGGCGTGGCGCTCCGC | Construction of orbS-C196A allele |
| Cysala2rev | GCGCCACGCGTCGAGGGCTTCCGCGCAGTGGCGCTCCGC | Construction of orbS-C199A allele |
| Cysala3for | TGCCTCGACGCGGCCCATCGCGGCGTC | Construction of orbS-C203A allele |
| Cysala4for | CGCGGCGTCGCCGCTCCGGTGTTCCTG | Construction of orbS-C209A allele |
| Cysalaallfor | CGCCGCGGAAGCCCTCGACGCGGCCCATCGCGGCGTCGC  CGCTCCGGTGTTCC | Construction of orbS-CtetraA allele |
| orbSfwd-HindIII | GCGCAAGCTTGTAACTCGGAATTTGACGGAGCAG | Construction of pBBR2-orbS_ΔP_ |
| orbS-rev-BamHI | GCGCGGATCCGTTTTTTTACCGCCGCCGCG | Construction of pBBR2-orbS_ΔP_ |
| orbSmut1 | CCGTGGGATCCACTGCGGATAACACGATGGC | Construction of Δ*orbS* mutant*. Bam*HI site |
| orbSmut2 | ATGGCACGCGTCGAGGCAGCCGAGGAACGGGTTTGC | Construction of Δ*orbS* mutant |
| orbSmut3 | GCAAACCCGTTCCTCGGCTGCCTCGACGCGTGCCAT | Construction of Δ*orbS* mutant |
| orbSmut4 | GCTAGAAGCTTACGCGGCGCAGCAGATCACG | Construction of Δ*orbS* and *orbS-CtetraA* mutants*.* HindIII site. |
| orbSfor6 | GCGCGGATCCATGGCCATGGCGGAAGTGCTC | Construction of *orbS-CtetraA* mutant*.* BamHI site. |
| orbS_500 fwd | GCGCGGATCCTTTCCGATCATGACGCAAGCC | Construction of *orbS-CtetraA* mutant |
| CtetraA_Rv | GGCTTGCGTCATGATCGGAAA | Construction of *orbS-CtetraA* mutant |
| orbS_check_fwd | TCCGTCCCAGTCACGATCGC | *ΔorbS* mutant screening |
| orbS_check_rv | CGAGCGTCGGCAACGAAAGC | *ΔorbS* mutant screening |
| pchEfor | GCGCGGTACCTGGATCCCGAGGAATTGACG | Construction of *pchE* mutant*.* KpnI site. |
| pchE_Rv2_NheI | TAGAGCTAGCATCGGCGTGCACGTC | Construction of *pchE* mutant*.* NheI site. |
| pchE_for2 | GTGTGGATCCGAACGACCCATACAAGGC | *ΔpchE* mutant screening |
| pchE_Rv2 | GCGCAAGCTTAGCGTCGAGATCGGTTGC | *ΔpchE* mutant screening |
| furoutFor | CTGGGCGTTCGCATCCTGGT | *fur*::Tp mutant screening |
| furoutRev | GGCAACGACTGGGCGATCGT | *fur*::Tp mutant screening |
| furcheck_fwd | AGCGGCCTTCGCCTTCTTGC | *Δfur*::Tp mutant screening |
| furcheck_Rv | GCGACGTGATCGCGCTCGAA | *Δfur*::Tp mutant screening |
| orbI_int_fwd | AACTGTTCGTCGCGCTGCTGGC | *orbI* qPCR |
| orbI_int_Rv | GACTGCTTCGCGCACCATCAGC | *orbI* qPCR. |
| qPCRrpoD_F | GTACGGCGAACTCCATGACC | qPCR internal control |
| qPCRrpoD_Rv | GATCGCGCACCTTGGATTTG | qPCR internal control |
| orbsfor7 | GCGCCATATGGCCATGGCGGAAGTGCTC | Cloning *orbS* alleles into pET14b. *Nde*I site. |
|  |  |  |
| **Oligo name** |  |  |
| orbHds6_oligofwd | AATTCGCGCTAAAAAAACGCGCCGGCCAACCGTCTATCAGACAGGAGA | Construction of pRLG770-P_orbH_ |
| orbHds6_oligoRv | AGCTTCTCCTGTCTGATAGACGGTTGGCCGGCGCGTTTTTTTAGCGCG | Construction of pRLG770-P_orbH_ |
| porbH-fwd-HindIII | AGCTTGCGGCGGCGGTAAAAAAACGCGCCGGCCAACCGTCTATCAGACAGGAGCG | Construction of pKAGd4-P_orbHds96_ |
| porbH-rev-BamHI | GATCCGCTCCTGTCTGATAGACGGTTGGCCGGCGCGTTTTTTTACCGCCGCCGCA | Construction of pKAGd4-P_orbHds96_ |

**Table S4. Steps used in BLItz assays**

| **Step** | **Name** | **Time (s)** | **Holder type^[1]^** | **Solution** |
| --- | --- | --- | --- | --- |
| 1 | Initial baseline | 60 | Tube | RNAP buffer |
| 2 | Baseline | 60 | Drop | RNAP buffer |
| 3 | Loading | 300 | Drop | 500 µg ml^-1^ His-tagged sigma factor in RNAP buffer ^[2]†^ |
| 4 | Baseline | 60 | Tube | RNAP buffer |
| 5 | Baseline | 60 | Drop | RNAP buffer (+ 25 µM Fe^2+^) ^[3] †^ |
| 6 | Association | 300 | Drop | Core RNAP (+ 25 µM Fe^2+^) ^[4] †^ |
| 7 | Dissociation | 300 | Tube | RNAP buffer |

^[1]^ The total volumes in each step were either 250 µl (tube) or 5 µl (drop)
^[2]^ 1.25 µl of sigma factor, 3.75 µl of RNAP buffer
^[3]^ 3.75 µl of RNAP buffer, 1.25 µl of either ddH_2_O or 100 µM Fe^2+^ solution
^[4]^ 3.75 µl of *E. coli* core RNAP (NEB) at varying concentrations, 1.25 µl of either ddH_2_O or 100 µM Fe^2+^ solution
^†^ Solutions were incubated at room temperature for approximately 10 min before assaying

| 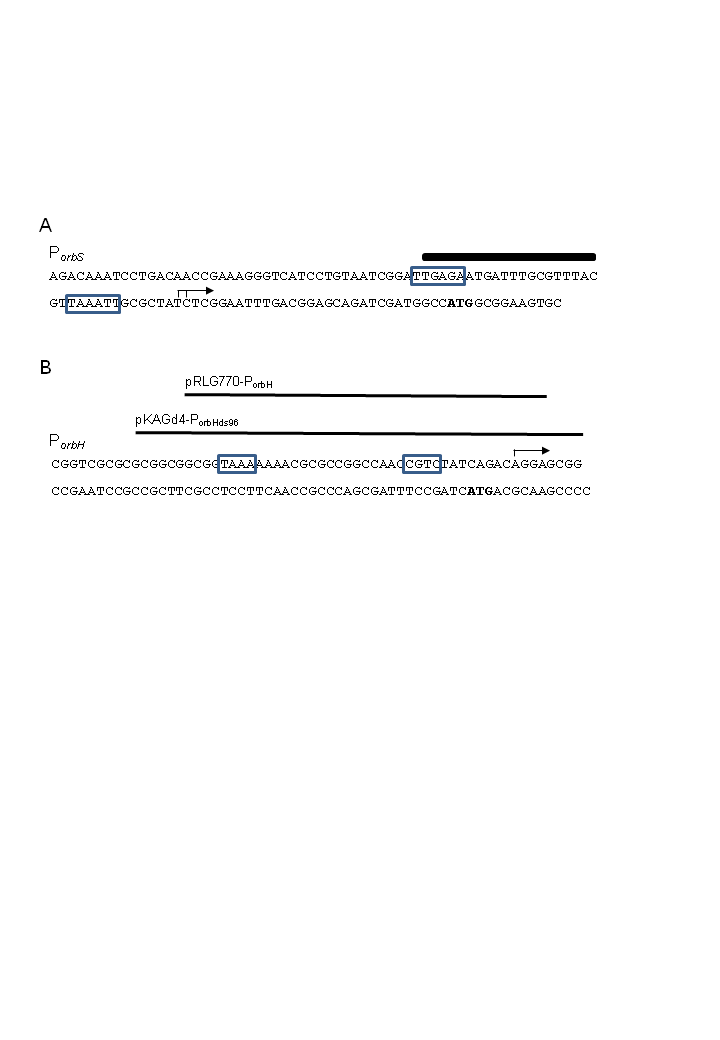 |
| --- |
| **Figure S1.** **Nucleotide sequences and features of the (A) *orbS* (*P_orbS_*) and (B) *orbH* (*P_orbH_*) promoters.** Transcription start sites are indicated by bent arrows. Conserved −35 and −10 sequences are enclosed in rectangles. The translation initiation codons (ATG) are shown in bold type. (A) The *P_orbS_* Fur box is indicated by the thick black line above the sequence. (B) The extent of the *P_orbH_* promoter sequences used to construct the *lacZ* reporter plasmid pKAGd4-*P*_orbHds96_ (-43 to +8) and the pRLG770-P_orbH_ plasmid (-37 to +5) used for *in vitro* transcription reactions are indicated by solid lines above the sequence. |

| 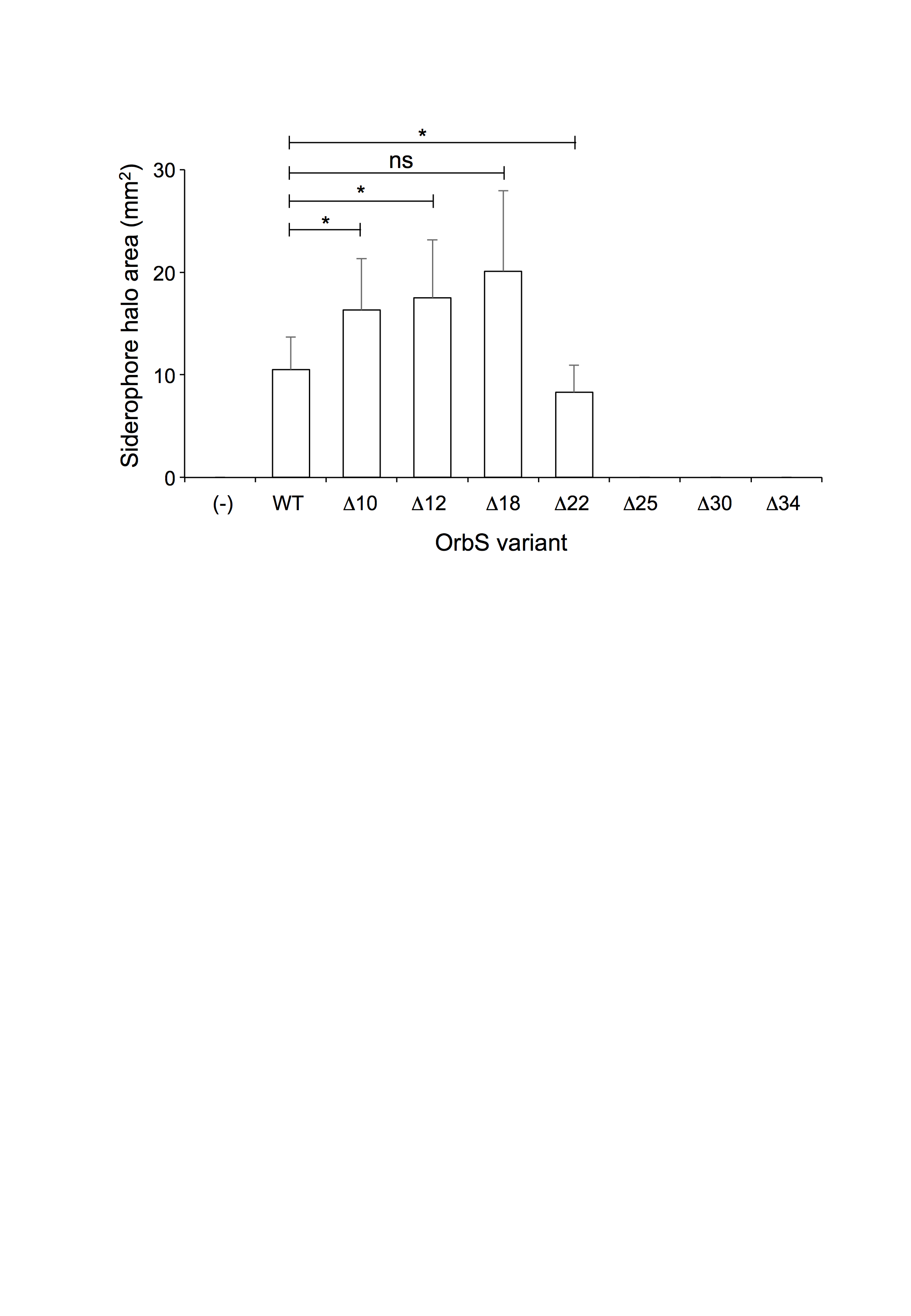 |
| --- |
| **Figure S2. Effect of C-terminal truncation of OrbS on ornibactin production.** Mean sizes of zones of iron depletion from the Fe-CAS-HDTMA complex due to ornibactin production by *B. cenocepacia* OM3 expressing C-terminally truncated OrbS derivatives (see Figure 3B). Error bars show standard deviation. *=*p*<0.05, ns= not significant following one-way ANOVA, with individual paired t tests. Significance is shown for the indicated OrbS variant compared to the wild-type (WT), n=3. |

| 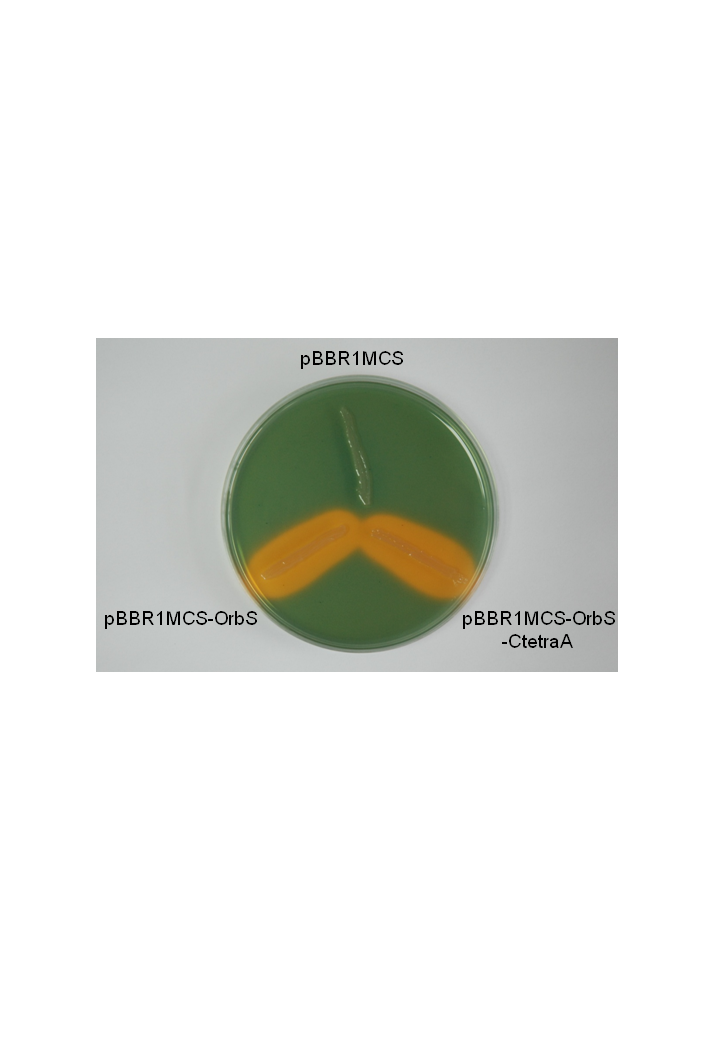 |
| --- |
| **Figure S3.** The *B. cenocepacia orbS* mutant, OM3, containing pBBR1MCS (top) or pBBR1MCS expressing *orbS* (bottom left) or *orbS-CtetraA* (bottom right) was streaked on standard CAS agar (containing 10 μM iron) and incubated at 37°C for 16 h. Ornibactin production is indicated by orange halos surrounding bacterial growth. |

| 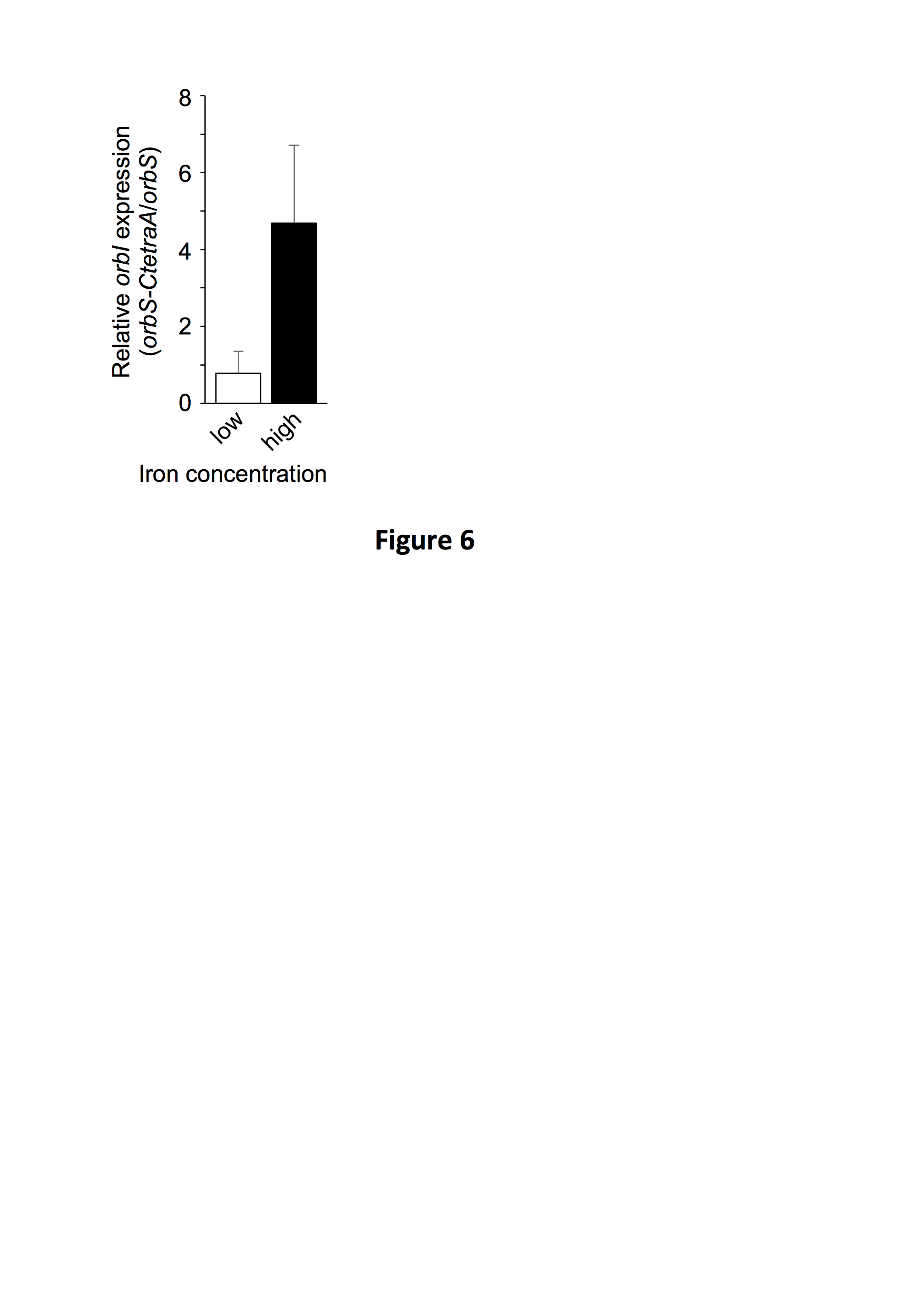 |
| --- |
| **Figure S4.** **The OrbS C-terminal cysteine residues are required for iron-responsive transcription of *orbI*.** qPCR analysis comparing *orbI* transcript abundance in a *B. cenocepacia* H111 *orbS-CtetraA* strain grown in high (50 μM FeCl_3_) and low iron 100 μM dipyridyl) conditions to a wild-type (OrbS*^+^*) strain. All strains contained *fur* and *pchE* null alleles. Relative expression was calculated using the 2^-ΔΔCT^ method and *rpoD* transcripts were used as an internal reference control. |

| 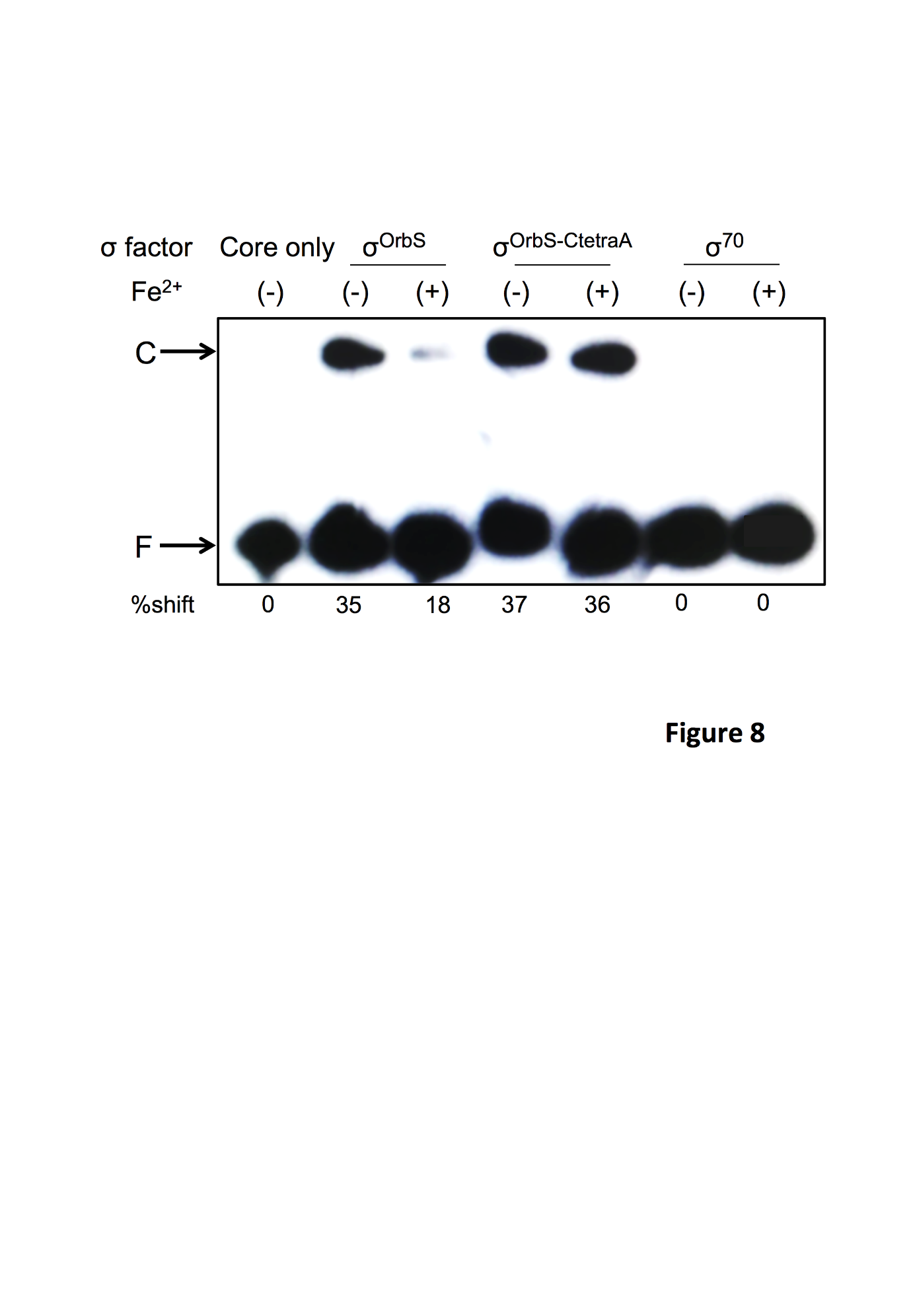 |
| --- |
| **Figure S5. Ferrous iron inhibits OrbS-dependent binding of RNA polymerase at the *orbH* promoter.** OrbS and OrbS-CtetraA were separately incubated with *E. coli* core RNAP in the presence or absence of 25 μM Fe(NH_4_)_2_(SO_4_)_2_ before the addition of labelled *P_orbH_* DNA probe and heparin. Following incubation at 37^o^C, samples were electrophoresed in a pre-run 4.5% polyacrylamide gel and the location of the labelled DNA probe was visualised on a phosphorimager following exposure of the gel to an imaging plate. The fraction of the probe that was shifted due to holo-RNAP binding (shown as %shift below each lane) was calculated by quantifying shifted OrbS-DNA probe complexes (C) and unshifted free DNA probe (F) using ImageJ software. σ^70^ holoRNAP was also included as a negative control. |

| 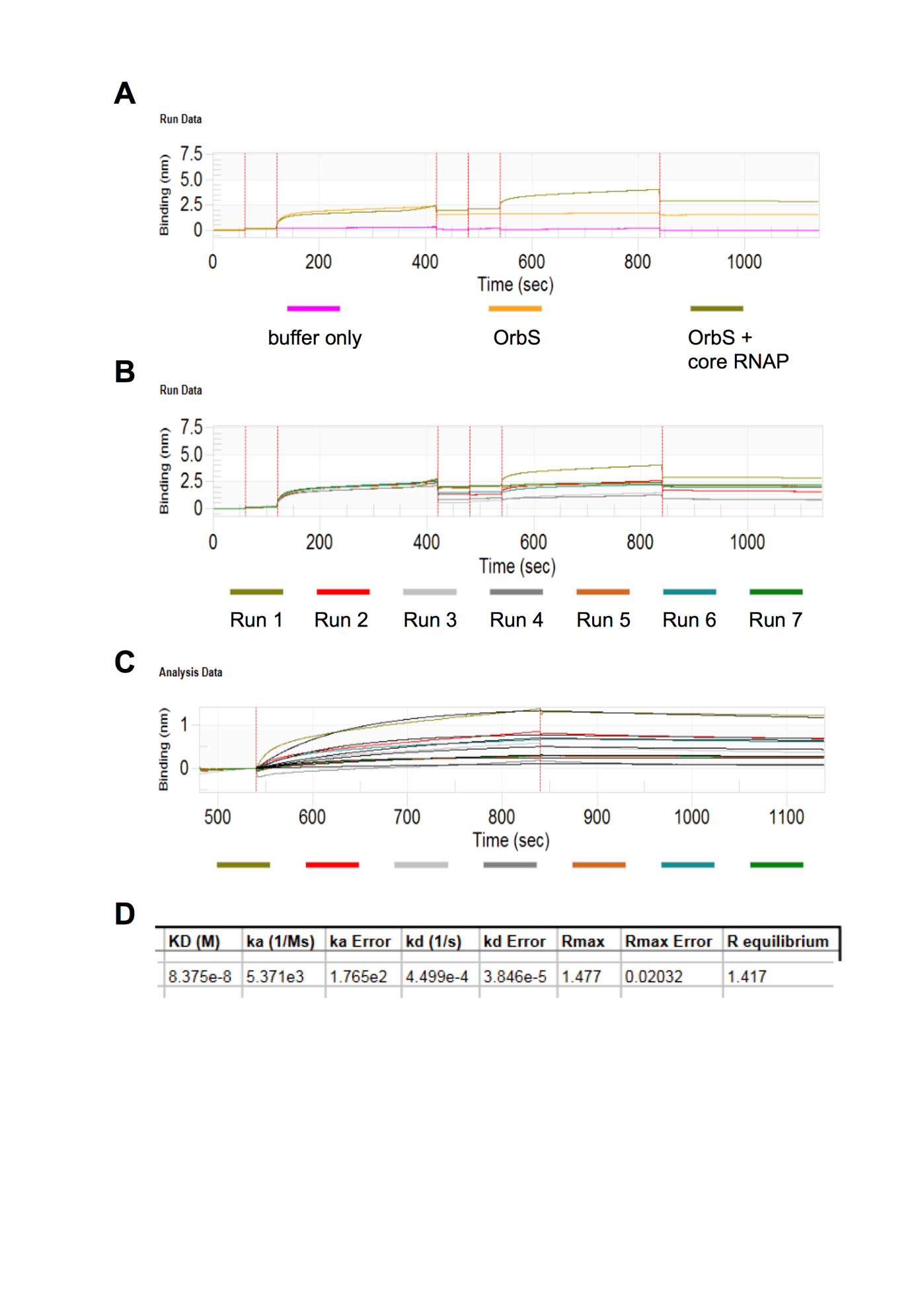 |
| --- |
| **Figure S6. Representative biolayer interferometry (BLItz) traces for interaction between immobilized OrbS and core *E. coli* RNAP in.**  A. Complete run cycle is shown. An association curve is observed for the OrbS-RNAP holoenzyme (550-850 s); B. Ensemble of experiments with different metal ion concentrations. C. Association (550-850 s) and disassociation (850-1150 s) phases from the experiments shown in (B) used to calculate the corresponding rate constants. D. Analysis output. |

| 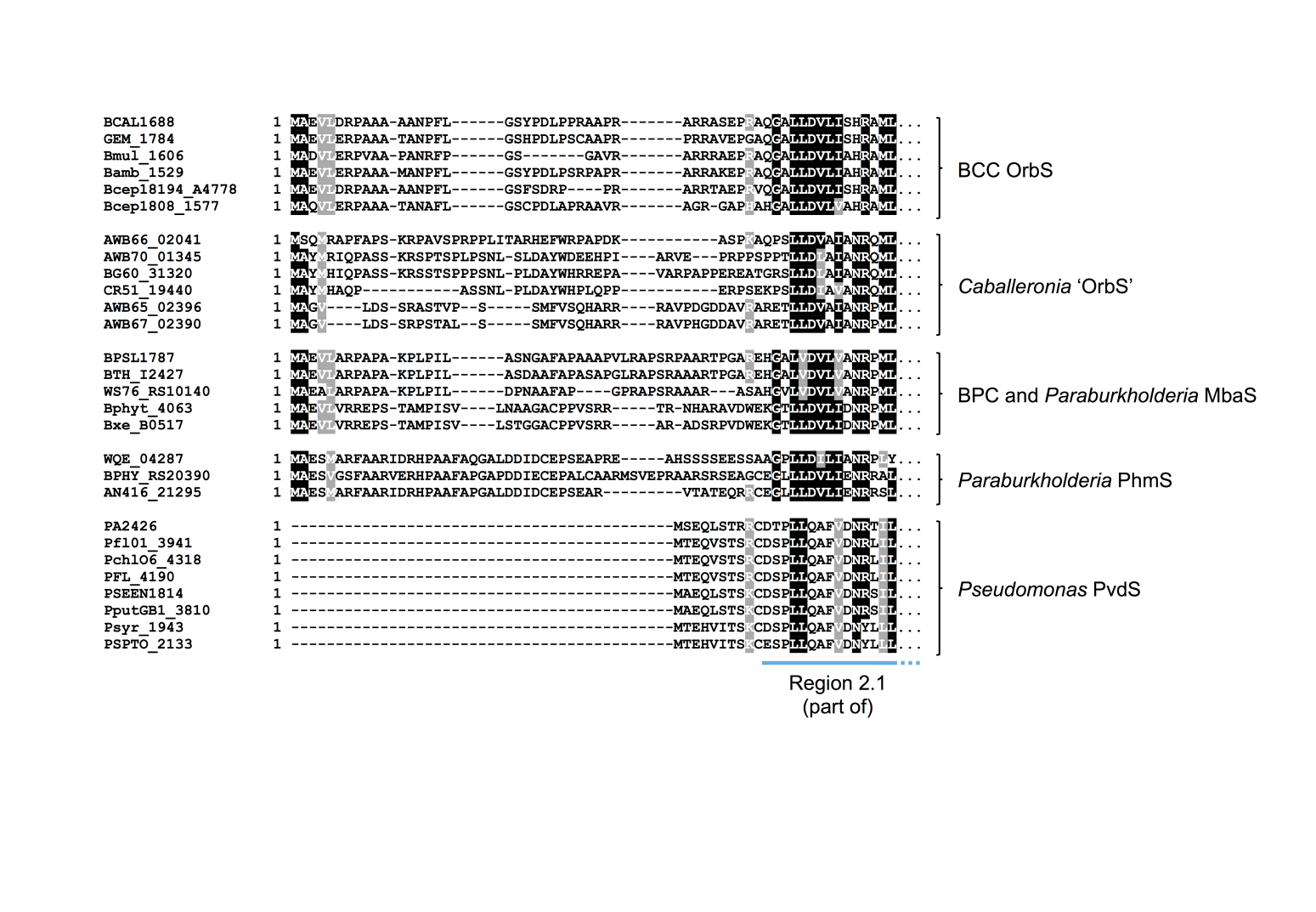 |
| --- |
| **Figure S7. Comparison of the N-terminal amino acid sequences of OrbS, MbaS and PhmS orthologues with the well-characterized *P. aeruginosa* IS ECF σ factor, PvdS, and its orthologues.** The sequence of residues 1-54 of *B. cenocepacia* OrbS (BCAL1688) is shown aligned with the corresponding region of representative OrbS, MbaS, PhmS and PvdS orthologues from other species (BCC, *B. cepacia* complex; BPC, *B. pseudomallei* complex), including *P. aeruginosa* PvdS (PA2426). The region shown includes ~15 residues of region 2.1 (indicated by the blue bar). Assignment of the sigma factor as OrbS in members of the genus *Caballeronia* is based on bioinformatics analysis of the OrbI and OrbJ NRPS orthologues which are predicted to generate ornibactin (results not shown). For other assignations, see Butt and Thomas (24). Amino acids that are identical at the corresponding position in ≥50% of sequences are shown in white font with black highlighting, whereas amino acids that are similar are shown with grey highlighting. |

**REFERENCES**

1. McKevitt,A.I., Bajaksouzian,S., Klinger,J.D. and Woods,D.E. (1989) Purification and characterization of an extracellular protease from *Pseudomonas cepacia*. Infect. Immun., **57,** 771-778.
2. Darling,P., Chan,M., Cox,A.D., and Sokol,P.A. (1998) Siderophore production by cystic fibrosis isolates of *Burkholderia cepacia*. Infect. Immun., **66**, 874–877.
3. Agnoli,K., Lowe,C.A., Farmer,K.L., Husnain,S.I. and Thomas,M.S. (2006) The ornibactin biosynthesis and transport genes of *Burkholderia cenocepacia* are regulated by an extracytoplasmic function sigma factor which is a part of the Fur regulon. J. Bacteriol., **188**, 3631-3644.
4. Gotschlich,A., Huber,B., Geisenberger,O., Tögl,A., Steidle,A., Riedel,K., Hill,P., Tümmler,B., Vandamme,P., Middleton,B., Camara,M., Williams,P., Hardman,A. and Eberl,L. (2001) Synthesis of multiple N-acylhomoserine lactones is wide-spread among the members of the *Burkholderia cepacia* complex. Syst. Appl. Microbiol., **24**, 1-14.
5. Yanisch-Perron,C., Vieira,J. and Messing,J. (1985) Improved M13 phage cloning vectors and host strains: nucleotide sequences of the M13mp18 and pUC19 vectors. Gene., **33**, 103-119.
6. Casadaban,M.J. and Cohen,S.N. (1980) Analysis of gene control signals by DNA fusion and cloning in *Escherichia coli*. J. Mol. Biol., **138**, 179-207.
7. Simon,R., Priefer,U. and Pühler,A. (1983) A broad host range mobilization system for in vivo genetic engineering: transposon mutagenesis in Gram negative bacteria. Biotechnology, **1**, 784-791.
8. Ferrières,L., Hémery,G., Nham,T., Guérout,A.-M., Mazel,D., Beloin,C. and Jean-Ghigo,J.-M. (2010) Silent mischief: Bacteriophage Mu insertions contaminate products of *Escherichia coli* random mutagenesis performed using suicidal transposon delivery plasmids mobilized by broad-host-range RP4 conjugative machinery. J. Bacteriol., **192**, 6418-6427.
9. Jackson,S.A., Fellows,B.J. and Fineran,P.C. (2020) Complete genome sequences of the *Escherichia coli* donor strains ST18 and MFDpir. Microbiol. Resour. Announc. **9**, e01014-20.
10. Herrero,M., de Lorenzo,V. and Timmis,K.N. (1990) Transposon vectors containing non-antibiotic resistance selection markers for cloning and stable chromosomal insertion of foreign genes in gram-negative bacteria. J. Bacteriol., **172**, 6557-6567.
11. Studier,F.W. and Moffatt,B.A. (1986) Use of bacteriophage T7 RNA polymerase to direct selective high-level expression of cloned genes. J. Mol. Biol., **189**, 113-130.
12. Carlioz,A. and Touati,D. (1986) Isolation of superoxide dismutase mutants in *Escherichia coli*: is superoxide dismutase necessary for aerobic life? EMBO J., **5**, 623-630.
13. Touati,D., Jacques,M., Tardat,B., Bouchard,L. and Despied,S. (1995) Lethal oxidative damage and mutagenesis are generated by iron in Δ*fur* mutants of *Escherichia coli*: protective role of superoxide dismutase. J. Bacteriol., **177**, 2305-2314.
14. Agnoli,K., Haldipurkar,S.S., Tang,Y., Butt,A.T. and Thomas,M.S. (2019) Distinct modes of promoter recognition by two iron starvation σ factors with overlapping promoter specificities. J. Bacteriol., **201**, e00507-18.
15. Kovach,M.E., Phillips,R.W., Elzer,P.H., Roop,R.M. and Peterson,K.M. (1994) pBBR1MCS: a broad-host-range cloning vector. Biotechniques., **16**, 800-802.
16. Lowe,C.A, Asghar,A.H, Shalom,G., Shaw,J.G. and Thomas,M.S. (2001) The

*Burkholderia cepacia fur* gene: co-localisation with *omlA* and absence of

regulation by iron. Microbiology, **147**, 1303-1314.

1. Kovach,M.E., Elzer,P.H., Hill,D.S., Robertson,G.T., Farris,M.A.,Roop,R.M. and Peterson,K.M. (1995) Four new derivatives of the broad-host-range cloning vector pBBR1MCS, carrying different antibiotic-resistance cassettes. Gene.,**166**, 175-176.
2. Ross,W., Thompson,J.F., Newlands,J.T. and Gourse,R.L. (1990) *E. coli* Fis protein activates ribosomal RNA transcription *in vitro* and *in vivo*. EMBO J., **9**, 3733-3742.
3. Shastri,S., Spiewak,H.L., Sofoluwe,A., Eidsvaag,V.A., Asghar,A.H., Pereira,T., Bull,E.H., Butt,A.T. and Thomas,M.S. (2017) An efficient system for the generation of marked genetic mutants in members of the genus *Burkholderia*. Plasmid., **89**, 49-56.
4. Dix,S.R., Owen,H.J., Sun,R., Ahmad,A., Shastri,S., Spiewak,H.L., Mosby,D.J., Harris,M.J., Batters,S.L., Brooker,T.A., Tzokov,S.B., Sedelnikova,S.E., Baker,P.J., Bullough,P.A., Rice,D.W. and Thomas,M.S. (2018) Structural insights into the function of type VI secretion system TssA subunits. Nat. Commun., **9**, 4765.
5. DeShazer,D. and Woods,D.E. (1996) Broad-host-range cloning and cassette vectors based on the R388 trimethoprim resistance gene. Biotechniques., **20**, 762-764.
6. Flannagan,R.S., Linn,T. and Valvano,M.A. (2008) A system for the construction of targeted unmarked gene deletions in the genus *Burkholderia*. Environ. Microbiol., **10**, 1652-1660.
7. Berrow,N.S., Alderton,D., Sainsbury,S., Nettleship,J., Assenberg,R., Rahman,N., Stuart,D.I., and Owens,R.J. (2007) A versatile ligation-independent cloning method suitable for high-throughput expression screening applications. Nucleic Acids Res., **35**, e45.
8. Butt,A.T. and Thomas,M.S. (2017) Iron acquisition mechanisms and their role in the virulence of *Burkholderia* sp*ecies. Front*. Cell. Infect. Microbiol., 7, 460.
